# Geometry-Based Design of a State-Dependent Process-Planning Space with Integrated Fixturing for 3 + 2-Axis Milling

**DOI:** 10.3390/ma19214444

**Published:** 2026-10-22

**Authors:** Sebastian Langula, Alexander Brosius

**Affiliations:** Chair of Forming and Machining Technology, Institute of Manufacturing, Dresden University of Technology, George-Bähr-Straße 3, 01069 Dresden, Germany; alexander.brosius@tu-dresden.de

**Keywords:** computer-aided process planning, 3 + 2-axis milling, operation sequencing, fixture planning

## Abstract

Automated operation-sequence planning for 3 + 2-axis computer numerical control (CNC) milling remains challenging because material removal changes both feature accessibility and the surfaces available for fixturing. This paper introduces an explicit state-transition formulation for constructing a solver-independent planning space from evolving workpiece geometry. The workpiece state combines volumetric material occupancy, surface-state availability, and completed machining features; it determines geometry-induced precedence and the availability of surfaces for workpiece fixturing. These relations are integrated with admissible tools, predefined tool-access directions, and machining-related cost contributions before optimisation begins. An Evolving Ant Colony System (EACS) algorithm then searches the resulting candidate space. Evaluation uses a 24-part benchmark with 58 EACS runs and 1000 random admissible samples per part. Functional and geometric verification covers precedence construction, 573 fixturing situations, and model-internal clamping-placement comparison. Compared with random admissible sampling, EACS achieved lower mean objective values for all 24 parts, with complete observed distributional separation for 23 parts. This comparison does not establish superiority over alternative optimisation algorithms. Four experienced CAM practitioners additionally assessed the generated plans for technological plausibility. The results demonstrate how evolving geometry can be converted into explicit state-dependent admissibility conditions, while mechanical fixture validation and scalability remain open limitations.

## 1. Introduction

Computer-aided process planning (CAPP) links product definition with manufacturing execution by transforming geometric and manufacturing information into operation sequences and resource assignments. Full automation remains challenging because operation sequencing, feature accessibility, tool selection, setup planning and workpiece state are mutually dependent decisions [1]. CAPP therefore forms an important integration layer between product definition and manufacturing execution [2]. In networked manufacturing environments, this integration extends to the coordination of process planning and scheduling [3]. A longstanding objective is the integration of computer-aided design (CAD), CAPP, computer-aided manufacturing (CAM), and computer numerical control (CNC) [4]. In machining, these dependencies are particularly pronounced because the workpiece geometry evolves during material removal. A feature that is initially inaccessible may become machinable after another volume has been removed, while the same state transition may expose new fixturing surfaces or eliminate previously available ones. Consequently, the set of admissible subsequent operations and resource assignments depends on the preceding manufacturing decisions.

Generative CAPP approaches therefore represent machining features, process alternatives and precedence constraints before determining an operation sequence. Khoshnevis et al. combined feature reasoning with state-space optimisation [5], while Šormaz and Khoshnevis represented feature interactions using precedence relations [6]. Graph-based metaheuristic approaches have subsequently been applied to search precedence-constrained process-planning spaces [7]. Learning-based process-route planning has been formulated as a Markov decision process using attributed adjacency graphs [8]. Subsequent work extended this approach through masking and Monte Carlo tree search to improve feasible-process exploration [9]. However, increasingly capable optimisation methods cannot compensate for planning relations or resource alternatives that are absent from the underlying problem representation.

The present study addresses this preceding planning-space construction problem. Geometric and manufacturing information is transformed into a state-dependent feasible planning space. Accessibility analysis of the evolving workpiece geometry yields mandatory precedence relations, while state-dependent surface analysis determines fixturing alternatives that become available or expire during manufacturing. Tool alternatives and predefined tool-access-direction (TAD) constraints are incorporated as additional resource information. Their integration defines the feasible planning space Ω before optimisation begins.

The subsequent optimisation layer contains two distinct tasks. An established EACS searches Ω to determine an optimised process plan comprising the operation sequence, tool assignments and fixturing assignments. The selected fixturing situations are subsequently resolved into concrete clamping-element positions and orientations by a geometric fixture-pose optimisation. Mechanical workholding additionally involves aspects such as force transmission, stiffness, deformation and thermal stability [10]. These effects, together with detailed machine- and clamp-level accessibility, are outside the present system boundary.

A precursor implementation of the underlying geometry-based planning procedure was presented in [11]. The present study extends this basis by introducing an explicit state-transition formulation, formal state-dependent resource admissibility and a solver-independent definition of the feasible planning space. Accordingly, the present study addresses three objectives: to formulate an explicit state-dependent planning model linking evolving geometry, precedence and fixturing availability; to integrate these relations with tool and predefined tool-access-direction information into a solver-independent feasible planning space; and to evaluate the resulting framework with respect to geometric consistency, planning-space search, computational performance and technological plausibility. Figure 1 provides an overview of the resulting framework, from geometric and manufacturing input data through state-dependent planning-space formalisation to optimisation and the resulting process plan and fixture configuration.

## 2. State of the Art

### 2.1. Search and Optimisation of Represented Process-Planning Spaces

Operation sequencing is a central combinatorial problem in generative CAPP. Early integrated systems combined feature reasoning with explicit process alternatives and search-based sequencing [5]. Subsequent approaches applied simulated annealing to minimise machine-, setup- and tool-related planning costs under precedence constraints [12]. Ma et al. used simulated annealing for process planning of prismatic parts [13]. Li et al. combined genetic algorithms with simulated annealing to balance global exploration and local improvement [14]. Liu et al. applied ant colony optimisation while jointly considering operation sequencing and manufacturing-resource decisions [15]. Dou et al. proposed a discrete particle-swarm formulation that directly generates feasible operation sequences [16].

Graph-based formulations remain widely used because they allow precedence relations and resource alternatives to be represented explicitly. Wang et al. modelled process planning as a directed graph and applied a two-stage ant colony optimisation method [7]. Su et al. introduced an edge-selection encoding strategy that preserves precedence feasibility while simultaneously optimising machine, tool-access-direction and cutting-tool choices [17]. Ha proposed an Evolving Ant Colony System for large integrated process-planning and scheduling problems with sequence-dependent setup times and tool-related constraints [18]. Milosevic et al. combined operation sequencing, manufacturing-resource selection, and setup planning within an operation-precedence graph [19].

The coupling between sequencing and setup-related decisions has also been addressed directly. Tseng and Liu considered machining sequence and fixturing setup concurrently in mill-turn machining [20]. Huang et al. combined a graph representation with a genetic algorithm for process-planning optimisation of prismatic parts [21]. Falih and Shammari proposed a hybrid constrained-permutation and genetic algorithm for process-planning problems [22].

Learning-based approaches provide a further search paradigm. Sugisawa et al. learned machining-sequence preferences from expert data using inverse reinforcement learning [23]. Zhang et al. formulated process-route planning as a Markov decision process based on attributed adjacency graphs [8]. In subsequent work, Zhang et al. improved feasible-process exploration through masking and Monte Carlo tree search [9].

Taken together, these approaches demonstrate that sophisticated mechanisms already exist for searching represented process-planning spaces. The unresolved issue addressed in the present work therefore lies one level earlier: the relevant planning constraints and alternatives must first be derived and represented from manufacturing information.

### 2.2. Geometry-Derived Planning Constraints and Evolving Workpiece States

Geometry-derived precedence is an established basis for operation sequencing. Šormaz and Khoshnevis derived feature-precedence networks from volumetric feature interactions and updated feature geometry during sequencing [6]. Liu and Wang transformed interacting-feature relationships into explicit machining-precedence relations [24]. Mokhtar and Xu distinguished local and global precedence relations for interacting 2.5D features [25]. Ding et al. combined model-based definition information with machining-feature recognition and process-route optimisation [26]. These studies show that geometric or feature-level relationships can be transformed into explicit sequence constraints before optimisation begins.

The manufacturing representation also determines which planning relations can be derived. Givehchi et al. proposed an enriched machining-feature concept for generic process sequencing [27]. Deja and Siemiatkowski linked feature-based process-plan generation with optimisation of part manufacture [28]. Liu et al. introduced a dynamic feature concept in which machining progress becomes part of the feature representation [29]. The present method assumes such a feature-resolved manufacturing task as input and focuses instead on deriving state-dependent planning information from the supplied geometry.

Volumetric representations have also been used to model intermediate workpiece states. Li et al. represented the in-process workpiece using a voxel model and encoded removal order through a subtraction field [30]. Šormaz et al. generated intermediate workpiece solids for selected process sequences and used them for downstream validation of tool, fixture, and machine interactions [31].

Dannen et al. provide the closest recent comparison. Their feature-independent approach voxelises intermediate workpiece states and applies reinforcement learning to both clamping-position identification and accessibility analysis [32]. These planning dimensions are, however, implemented as separate learned decision models. Their work therefore demonstrates that both accessibility and clamping can depend on evolving geometry while leaving their joint representation within one planning model unresolved.

The literature thus establishes geometry-derived precedence, evolving material states and state-dependent accessibility as individual capabilities. Mandatory sequencing relations alone, however, do not define the resource alternatives required for a complete process-planning problem. The next question is therefore how setup and fixturing alternatives can be linked to the same evolving manufacturing state.

### 2.3. Geometry- and Feature-Based Setup and Fixture Planning

Setup planning and fixture planning have been integrated at different abstraction levels. Kannan and Wright combined setup sequencing with fixture planning for 2.5D machining using standard fixturing elements [33]. Manafi and Nategh formulated concurrent setup and fixture planning using geometric and technological rules [34]. Zhang et al. developed a STEP-NC-compliant setup-planning method that groups features according to tool-approach-direction rules and determines locating datums within the planning process [35].

Computer-aided fixture design covers a broad range of representation, synthesis and verification tasks. Cecil reviewed core CAFD functions and their integration with CAD and process planning [36]. Bi and Zhang framed flexible fixture automation as an integration problem spanning representation, synthesis and verification [37]. Pehlivan and Summers analysed the information required across fixture-design activities [38]. Wang et al. reviewed locating, clamping and fixture-verification methods [39]. A systematic review confirms that automated fixture planning remains distributed across heterogeneous geometric, rule-based and optimisation approaches [40]. Bi et al. additionally highlighted the increasing role of artificial intelligence in fixture-design automation [41].

More recent setup-planning approaches further integrate process-planning information. Wakhare et al. combined feature precedence, tool-approach directions, and tolerance relationships within an automated setup-planning expert system [42]. Their approach substantially integrates feature reasoning, setup formation and sequencing, but does not derive fixture alternatives from an explicitly evolving material state.

Fixture planning spans both geometric and physical levels. Möhring et al. emphasise that workpiece clamping affects stiffness, thermal stability, positioning accuracy, and force transmission [10]. At the geometric level, interference checking and collision-free fixture assembly are established tasks. Hu and Rong developed an automated interference-checking method [43]. Anantharajan et al. addressed interference-free modular fixture assembly [44]. Physical workholding, however, additionally depends on restraint, machining loads, clamping forces, and workpiece deformation. DeMeter analysed fixture restraint under surface contact [45]. Meyer and Liou investigated dynamic machining loads [46]. Li et al. derived reaction and minimum-clamping-force conditions [47]. Finite-element approaches have also been used to analyse flexible fixture behaviour [48]. Workpiece deformation under fixturing has likewise been studied explicitly [49].

The present work deliberately addresses the geometric planning level. Candidate fixturing situations are derived from state-dependent workpiece surfaces and filtered using geometric criteria, while mechanical fixture behaviour remains outside the current model. Taken together, the reviewed approaches cover most individual elements required for integrated process planning, but differ in whether geometry-derived precedence, intermediate material states, fixturing and sequencing are represented jointly. Table 1 summarises these differences with respect to the present research question.

Table 1 is intended as a qualitative comparison of capabilities relevant to the present research question rather than as an exhaustive taxonomy. It shows that the required individual capabilities are established, whereas their joint state-dependent representation remains fragmented across geometry reasoning, setup planning and optimisation.

### 2.4. Previous Integrated Framework and Research Gap

The geometry-based planning procedure underlying the present study was originally developed and implemented in [11]. The precursor work already introduced geometry-derived precedence relations, intermediate workpiece states, geometry-based fixturing alternatives, and their integration into operation-sequence optimisation. It also established the original 24-part benchmark and implemented an EACS for searching the resulting operation-planning space. The present study builds upon these established methods and focuses on their explicit mathematical formalisation. Table 2 summarises the distinction between the precursor work and the contributions of the present study.

The principal contribution of the present study is therefore not the introduction of the individual geometric planning procedures, but their consolidation into an explicit state-dependent planning-space formulation. The evolving workpiece state combines volumetric material occupancy, surface-state availability, and completed machining features. Its transitions determine the conditions under which subsequent operations and fixturing alternatives become admissible or cease to be available. By integrating these conditions with geometry-induced precedence and manufacturing-resource constraints, the resulting formulation separates the representation of feasible-process plans from the optimisation method used to search them.

The literature reviewed in Section 2.1, Section 2.2 and Section 2.3 demonstrates that geometry-derived precedence, intermediate workpiece states, fixture planning, and optimisation have been addressed through different modelling approaches. Recent studies also demonstrate state-dependent accessibility and clamping decisions [32]. However, within the literature reviewed here, their explicit integration into a unified, solver-independent feasible planning-space formulation for 3 + 2-axis milling remains insufficiently addressed. This observation is consistent with the broader challenge of representing interactions between planning decisions in automated process planning [50].

Accordingly, the present study addresses this formalisation gap by defining the evolving workpiece state, its transitions, and the associated state-dependent admissibility conditions within a common mathematical framework. The evaluation further examines the consistency and behaviour of this representation using the previously established benchmark, without claiming an independent experimental data basis. Section 3 develops the resulting formulation.

## 3. Proposed Methodology

The proposed methodology formalises automated process planning for indexed 3 + 2-axis milling as the construction and subsequent search of a geometry-derived feasible planning space. The following sections define the evolving workpiece state, geometry-derived precedence constraints and state-dependent resource alternatives that determine this space, followed by its optimisation.

### 3.1. Scope, Assumptions and Input Data

The methodology addresses automated process planning for indexed 3 + 2-axis milling. Its output is an optimised operation sequence with admissible tool and fixturing assignments, followed by geometric instantiation of the selected fixturing situations as concrete clamping-element poses. Detailed numerical-control (NC) toolpaths and continuous tool motions are not generated. Machine-workspace limits, complete spindle-head kinematics and detailed tool-holder trajectories are likewise not modelled explicitly.

The following terminology distinguishes the different levels of fixturing representation. A fixturing situation comprises a geometrically admissible pair of opposing contact areas and a corresponding workpiece orientation. A resource configuration combines an admissible cutting tool with a fixturing situation for a machining feature. A setup refers to consecutive machining operations performed without changing the selected fixturing situation. After operation-sequence optimisation, a clamping-element pose specifies the position and in-plane orientation of a standardised clamping element within the selected contact regions. The term fixture configuration refers to the resulting geometric realisation of the selected fixturing situation rather than an additional independent planning variable.

The method starts from a feature-resolved manufacturing task. Automatic feature recognition and alternative feature decomposition are outside the present scope; established approaches for manufacturing-feature recognition are available in the literature [51]. The supplied feature set comprises roughing features (RFs) and finishing features (FFs). Let *F* denote the complete machining-feature set, with *F_R_* and *F_F_* denoting the roughing- and finishing-feature subsets, respectively:

(1) $$F=F_{R}\cup F_{F}.$$

Roughing-feature preparation follows the octree-based approach of Erler [52]. Starting from the stock and finished-part geometries, material-removal volumes are represented volumetrically and segmented into machining-related regions. The resulting roughing features, together with their associated tool information and predefined tool-access directions, are treated as input to the present planning method. Finishing-feature preparation follows the surface-based approach of Erler and Brosius [53]. Finishing features are represented as regions of the finished-part surface and are supplied together with their associated manufacturing information. Thus, both roughing and finishing features enter the planning method with one predefined tool-access direction. The present method consequently optimises one supplied manufacturing interpretation rather than alternative feature decompositions or tool-access directions. This restriction directly affects the feasible planning space, as alternative TADs may change the geometry-derived precedence relations and the compatibility of machining features with candidate workpiece orientations, thereby modifying the admissible fixturing alternatives. The extent of this restriction cannot be quantified from the present benchmark, which specifies only one TAD per feature.

Each machining feature *f_i_* ∈ *F* is specified by its geometry and position in the common workpiece coordinate system, one predefined tool-access direction *d_i_*, a set *T_i_* of admissible cutting tools, and a machining-related cost contribution for each admissible tool. These quantities are supplied as input and are not re-derived during operation-sequence planning. In particular, *d_i_* is a fixed feature constraint, whereas the cutting tool is selected from *T_i_* during planning. Fixturing alternatives are not supplied as predefined resources; they are derived from the evolving workpiece geometry in Section 3.4. The resulting system boundary is summarised in Table 3.

### 3.2. Geometric Representation and Evolving Workpiece States

The geometry-based planning procedure requires complementary surface and volumetric representations. The finished-part surface is represented by a stereolithography (STL) mesh, while a sparse voxel-octree representation provides the discretised material occupancy required for volumetric reasoning. Roughing features are represented as material-removal volumes and finishing features as surface regions of the finished-part mesh. These representations are summarised in Figure 2.

All geometric representations share the same workpiece coordinate system and orientation. Finishing features are mapped to the common volumetric grid when required for volumetric accessibility analysis. The initial workpiece occupancy is represented as the union of the finished-part volume and the material-removal volumes of all roughing features:

(2) $$V_{0}=V_{P}\cup \underset{f_{i}\in F_{R}}{⋃}V_{i}^{R},$$

where *V_P_* denotes the finished-part volume and $V_{i}^{R}$ the material-removal volume associated with roughing feature *f_i_*. Let *D_k_* ⊆ *F* denote the set of machining features completed after *k* executed operations. Thus, *F_R_*∖*D_k_* contains the roughing features not yet completed. The occupied workpiece volume in state *k* is then

(3) $$V_{k}=V_{P}\cup \underset{f_{i}\in F_{R}\setminus D_{k}}{⋃}V_{i}^{R}.$$

Equation (3) assumes non-overlapping roughing-feature removal volumes. If two features shared the same voxel, executing the first would not remove that voxel from the union of the remaining feature volumes. Overlapping volumetric removal operations are therefore outside the present formulation.

Equation (3) describes only the volumetric component of the evolving workpiece state. Process planning additionally requires the current condition of relevant surface entities. The workpiece state after *k* executed operations is therefore defined as

(4) $$X_{k}=(V_{k},\Sigma_{k},D_{k}),$$

where *V_k_* denotes the current volumetric material occupancy, Σ*_k_* the current surface-state representation, and *D_k_* the set of completed machining features. Execution of any machining feature *f_i_* updates the completed-feature set according to *D_k_*_+1_ = *D_k_* ∪ {*f_i_*}. Roughing operations update both *V_k_* and Σ*_k_* by removing material and exposing or removing surface entities. Finishing operations do not introduce an additional volumetric removal term in the present abstraction; instead, they change the state of the corresponding surface entities from pre-finishing to finished. The surface-state representation therefore distinguishes roughing-induced surfaces, surfaces awaiting finishing, finished surfaces and non-feature surfaces. For example, a surface exposed by roughing may initially be available for fixturing. Subsequent finishing changes its state without modifying the volumetric representation, potentially invalidating configurations requiring the pre-finishing surface while enabling configurations based on the finished surface.

Execution of machining feature *f_i_* induces the state transition

(5) $$X_{k+1}=\tau (X_{k},f_{i}),$$

where *τ* updates *D_k_* and, depending on the feature type, the volumetric and surface-state components. This representation makes the creation and expiration of potential contact surfaces explicit as manufacturing progresses.

The evolving workpiece state provides the geometric basis for the planning-information derivations that follow. Section 3.3 derives mandatory geometry-induced precedence relations. Section 3.4 derives geometry-based fixturing candidates and the surface-state conditions associated with their use. Section 3.5 subsequently combines these components with manufacturing-resource information to determine state-dependent admissibility and define the feasible planning space.

### 3.3. Geometry-Based Derivation of Machining Precedence Constraints

The first geometry-based planning step derives mandatory precedence constraints by evaluating geometric blocking along the tool-approach paths defined by the predefined tool-access directions. All machining features are represented on the common volumetric grid introduced in Section 3.2. For each feature *f_i_*, let *Q_i_* denote the set of volumetric cells associated with the feature and *d_i_* its predefined unit tool-access direction. For every cell *q* ∈ *Q_i_*, an accessibility ray is cast from the cell centre *c_q_* according to

(6) $$r_{iq}(\lambda )=c_{q}-\lambda d_{i},\;\lambda \in R_{>0}.$$

If an accessibility ray intersects occupied cells assigned to another machining feature *f_j_*, feature *f_j_* geometrically blocks access to *f_i_*. This intersection induces the mandatory precedence relation

(7) $$f_{j}≺f_{i},$$

indicating that *f_j_* must be completed before *f_i_* becomes geometrically accessible. Algorithm 1 applies this accessibility test to all feature cells and returns the resulting set of unique blocking relations.
**Algorithm 1.** Voxel-based accessibility analysis for deriving mandatory precedence relations.deriveBlockingRelations(features: Set<Feature>): Set<(Feature, Feature)>1
blockingRelations:= ∅2
**for** f_i ∈ features do3│   Q_i: Set<Voxel>:= voxels(f_i)4│   **for** q ∈ Q_i do5│       r:= Ray(center(q), -d_i)6│       H:= intersectedFeatures(r) \ {f_i}7│       **for** f_j ∈ H do8│           blockingRelations:= blockingRelations ∪ {(f_j, f_i)}  // f_j blocks f_i9│       **end**10│   **end**11
**end**12
**return** blockingRelations

Figure 3 illustrates the derivation for a representative voxel configuration. In Figure 3a, accessibility rays are evaluated opposite to the predefined tool-access directions. RF1 blocks access to RF2 and FF2, whereas RF2 blocks access to FF1. The resulting blocking relations are represented as directed edges in the precedence graph shown in Figure 3b.

The precedence graph is defined as

(8) $$G_{P}=(V,E_{P}),$$

where *V* = {*v_i_*∣*f_i_* ∈ *F*} contains one vertex for each machining feature and *E_P_* contains the corresponding directed precedence edges. Each precedence relation *f_j_*≺*f_i_* is represented by a directed edge (*v_j_*,*v_i_*) ∈ *E_P_*. The graph therefore represents the mandatory ordering constraints induced by geometric accessibility without prescribing a unique machining sequence.

Because accessibility is evaluated from discrete voxel centres, sufficiently narrow obstructions may remain undetected if they lie between neighbouring accessibility rays. This sampling limitation is quantified in Section 4.2.1 and evaluated in Section 5.1. The resulting precedence graph *G_P_* constitutes the first geometry-derived constraint component of the planning problem. State-dependent fixturing alternatives are derived in the following Section 3.4.

### 3.4. Geometry-Based Derivation of Fixturing Situations

The second geometry-based derivation step identifies the fixturing situations available in each workpiece state *X_k_*. Rather than assuming predefined fixturing alternatives, the proposed method derives candidate fixturing situations directly from the surface-state entities available in Σ*_k_*. Relevant surfaces may originate from the initial stock, roughing-induced intermediate states, finishing surfaces prior to machining, and the corresponding finished surface states after finishing.

The derivation follows a coarse-to-fine procedure, as summarised in Figure 4. State-dependent surface entities are first converted into potential fixturing areas (Section 3.4.1). These areas are subsequently evaluated according to their geometric suitability (Section 3.4.2), combined into opposing surface pairs and checked for compatibility with admissible table orientations (Section 3.4.3), and finally filtered for volumetric collisions (Section 3.4.4). The resulting set of state-dependent, geometrically admissible fixturing situations is subsequently integrated with the precedence structure.

#### 3.4.1. State-Dependent Surface Segments and Potential Fixturing Areas

Finished-part surfaces are obtained directly from the STL model, whereas stock and roughing-induced intermediate surfaces are extracted from the volumetric component *V_k_* by identifying boundaries between occupied and unoccupied cells. Combined with the surface-state information Σ*_k_*, these surfaces represent the contact geometry available in the current workpiece state *X_k_*. Spatially adjacent boundary elements satisfying the prescribed coplanarity condition are merged into coherent surface segments. Segments that are not eligible for contact generation are excluded from further processing. Figure 5 illustrates the transition from the grid-based surface representation to the clustered surface regions used for the subsequent derivation of potential fixturing areas.

Each retained surface segment *S* is discretised in its local orientation plane using a regular two-dimensional grid *G_S_*. The grid-cell edge length *L_g_* defines the spatial resolution of this representation. For each grid cell *g* ∈ *G_S_*, a visibility configuration *M*[*g*] is derived from the relevant workpiece geometry along the segment normal. The visibility configuration encodes the local geometric obstruction state relevant to fixture contact at grid cell *g*. Neighbouring grid cells with identical visibility configurations are subsequently grouped into connected components. Each connected component constitutes one potential fixturing area *P_i_*. Algorithm 2 summarises this derivation.
**Algorithm 2.** Derivation of state-dependent potential fixturing areas.derivePotentialFixturingAreas(X_k_: State, L_g: Length): Set<Area>1
segments:= eligibleSegments(clusterCoplanarSurfaces(extractAvailableSurfaces(X_k_)))2
areas: Set<Area>:= ∅3
**for** S ∈ segments do4│   G_S:= projectToGrid(S, L_g)5│   **for** g ∈ G_S do6│      M[g]:= visibilityConfiguration(g, normal(S), X_k_)7│   **end**
8│   **for** C ∈ connectedComponents(G_S, M) do9│      areas:= areas ∪ {createArea(S, C)}10│   **end**11
**end**12
**return** areas

Connected-component analysis groups only spatially adjacent grid cells with identical visibility configurations. Consequently, geometrically disconnected regions remain separate potential fixturing areas even if they share the same visibility configuration. Because both the available surface entities and their visibility depend on *X_k_*, the resulting set of potential fixturing areas is state dependent. Roughing operations can expose or remove contact regions, whereas finishing operations can modify their surface state without necessarily changing the volumetric component *V_k_*. The grid-cell edge length *L_g_* used in the evaluation is reported in Section 4.1, and the associated discretisation effect is quantified in Section 4.2.1. The resulting areas are subsequently evaluated according to the suitability criteria introduced.

#### 3.4.2. Fixturing Suitability Map

The local geometric suitability of each grid cell is evaluated using two heuristic criteria. The first criterion favours cells located farther from the boundary of the corresponding potential fixturing area, thereby preferring locations with a larger surrounding contact region. For each grid-cell centre *c*, the distance to the area boundary is approximated by casting *N_B_* uniformly distributed in-plane search rays and retaining the smallest detected intersection distance. Increasing *N_B_* improves the angular resolution of this approximation at the expense of additional geometric evaluations.

The second criterion favours cells located closer to the centroid *s* of the material-removal volume represented by the difference octree. Figure 6 illustrates both geometric criteria for an individual grid cell.

Let $d_{i}^{B}\left(c\right)$ denote the in-plane distance from grid-cell centre *c* to the first intersection with the boundary of potential fixturing area *P_i_* along search direction *i*. The approximated boundary distance is obtained as

(9) $$d^{B}\left(c\right)=\underset{i=1,\ldots ,N_{B}}{\min}d_{i}^{B}\left(c\right).$$

Let *L*_stock_ denote the diagonal length of the initial-stock bounding box. The unscaled boundary-distance score is then defined as

(10) $$S_{\mathrm{edge}}^{\mathrm{raw}}\left(c\right)=\frac{d^{B}\left(c\right)}{L_{\mathrm{stock}}}.$$

The corresponding centroid-distance score is defined as

(11) $$S_{\mathrm{vol}}^{\mathrm{raw}}\left(c\right)=1-\frac{∥c-s∥}{L_{\mathrm{stock}}}.$$

Thus, larger values of $S_{\mathrm{edge}}^{\mathrm{raw}}$ correspond to locations farther from the area boundary, whereas larger values of $S_{\mathrm{vol}}^{\mathrm{raw}}$ correspond to locations closer to the material-removal-volume centroid.

Before the two criteria are combined, their raw score fields are independently linearly rescaled to the interval [0,1] over all evaluated grid cells. For either criterion, the implemented transformation is

(12) $$\hat{S}=\frac{S^{\mathrm{raw}}-S_{\min}^{\mathrm{raw}}}{S_{\max}^{\mathrm{raw}}-S_{\min}^{\mathrm{raw}}},$$

where $S_{\min}^{\mathrm{raw}}$ and $S_{\max}^{\mathrm{raw}}$ denote the minimum and maximum raw values of the corresponding criterion across the evaluated fixturing areas. For a uniform score field ($S_{\max}^{\mathrm{raw}}\;=\;S_{\min}^{\mathrm{raw}}$), the normalised values are set to 0.5, avoiding division by zero without introducing artificial spatial preferences. The resulting normalised scores are denoted by ${\hat{S}}_{\mathrm{edge}}$ and ${\hat{S}}_{\mathrm{vol}}$.

The combined suitability score of an individual grid cell is then

(13) $$S_{\mathrm{cell}}=w_{1}{\hat{S}}_{\mathrm{edge}}+w_{2}{\hat{S}}_{\mathrm{vol}},w_{1}+w_{2}=1.$$

The weighting parameters *w*_1_ and *w*_2_ determine the relative influence of the boundary-distance and centroid-distance criteria, respectively. Increasing either weight shifts the resulting suitability map towards cells favoured by the corresponding geometric criterion. The weights therefore represent heuristic planning preferences rather than parameters of a mechanically validated fixture-stability model.

The suitability of a potential fixturing area *P_i_* is obtained as the arithmetic mean of the cell-level suitability scores within the area:

(14) $$S\left(P_{i}\right)=\frac{1}{|P_{i}|}\sum_{g\in P_{i}}S_{\mathrm{cell}}\left(g\right).$$

The values of *N_B_*, *w*_1_, and *w*_2_ used in the evaluation are specified in Section 4.1. The resulting area-level suitability score *S*(*P_i_*) is subsequently used for ranking candidate contact configurations.

#### 3.4.3. Opposing-Surface Pairing and Table-Orientation Compatibility

Candidate contact configurations are derived by pairing potential fixturing areas with approximately opposing surface normals. Let *n_i_* and *n_j_* denote the unit normals of two potential fixturing areas *P_i_* and *P_j_*, respectively. A pair is considered only if

(15) $$∥n_{i}+n_{j}∥\leq \varepsilon_{n},$$

where *ε_n_* defines the admissible tolerance in the vector-sum norm relative to exactly opposing unit surface normals. The corresponding maximum angular deviation from exact opposition is *δ*_max_ = 2arcsin(*ε_n_*/2). Smaller values of *ε_n_* therefore restrict the candidate set to more nearly opposing surfaces.

In addition to normal opposition, candidate pairs must provide sufficient projected overlap and geometric separation. Let *A*_ov_(*P_i_*,*P_j_*) denote the projected overlap area of both contact regions. A candidate pair is retained only if

(16) $$A_{\mathrm{ov}}\left(P_{i},P_{j}\right)\geq A_{\min},$$

where *A*_min_ denotes the minimum geometric contact footprint required for subsequent clamping-element assignment.

The separation between both supporting planes is evaluated relative to the workpiece extent in their common normal direction. For a candidate pair *P_i_* and *P_j_*, the corresponding unit direction is defined as

(17) $$n=\frac{n_{i}-n_{j}}{∥n_{i}-n_{j}∥}.$$

The workpiece extent along this direction is obtained from the axis-aligned bounding box (AABB) as

(18) $$L_{n}=\underset{x\in \mathrm{AABB}}{\max}\left(n\cdot x\right)-\underset{x\in \mathrm{AABB}}{\min}\left(n\cdot x\right).$$

A surface pair is retained if

(19) $$d\left(P_{i},P_{j}\right)>\lambda_{d}L_{n},$$

where *d*(*P_i_*,*P_j_*) denotes the plane-to-plane separation along the common normal direction and *λ_d_* controls the required separation relative to the workpiece extent. Increasing *λ_d_* favours more widely separated contact surfaces and therefore geometrically broader support configurations. This criterion is heuristic and does not represent a mechanically validated fixture-stability threshold. The values of *ε_n_*, *A*_min_, and *λ_d_* used in the evaluation are specified in Section 4.1. The retained surface pairs are ranked according to their plane separation, projected overlap, and geometric suitability. Let $d_{\mathrm{ij}}^{*}$ denote the normalised plane separation of contact areas *P_i_* and *P_j_*, and let $A_{\mathrm{ij}}^{*}$ denote their normalised projected overlap area. The resulting ranking score is defined as

(20) $$\Phi_{\mathrm{ij}}={(d}_{\mathrm{ij}}^{*})\cdot {(A}_{\mathrm{ij}}^{*})\cdot \left(\frac{S\left(P_{i}\right)+S\left(P_{j}\right)}{2}\right).$$

The normalised plane separation is defined as

(21) $$d_{\mathrm{ij}}^{*}=\frac{d\left(P_{i},P_{j}\right)}{L_{n}},$$

where *L_n_* denotes the stock-AABB extent along the common normal direction, as defined in Equation (18). The normalised projected overlap is defined as

(22) $$A_{\mathrm{ij}}^{*}=\frac{A_{\mathrm{ov}}\left(P_{i},P_{j}\right)}{A_{\mathrm{AABB},\max}},$$

where *A*_AABB,max_ denotes the largest face area of the stock-AABB,

(23) $$A_{\mathrm{AABB},\max}=\max\left(L_{x}L_{y},\;L_{x}L_{z},\;L_{y}L_{z}\right)$$

with *L_x_*, *L_y_*, and *L_z_* denoting the stock-AABB edge lengths. The indices *i* and *j* identify the two contact areas of the pair. The three geometric contributions in Equation (20) are combined multiplicatively so that a low value of plane separation, projected overlap, or local surface suitability directly reduces the overall ranking score. No additional weighting parameters are introduced at this stage. The score is used solely to rank geometrically suitable contact configurations and does not constitute a mechanical fixture-stability metric. Possible workpiece orientations relative to the machine table are subsequently evaluated from a finite set of candidate table-normal directions. The angular discretisation is controlled by the sampling parameters *N*_tab,1_ and *N*_tab,2_, which define the resolution of the two angular dimensions used to generate the candidate orientation space. Increasing either parameter increases the number of representable workpiece orientations but also the number of candidate configurations evaluated during preprocessing. Figure 7 illustrates the discrete table-orientation sampling and its relation to the predefined tool-access directions.

Let *r_m_* denote the outward unit normal of candidate table orientation *m*, as illustrated in Figure 7, and let *d_i_* denote the predefined tool-access direction of machining feature *f_i_*, pointing towards the workpiece. With this vector convention, the predefined tool-access direction is compatible with candidate table orientation *m* if

(24) $$d_{i}\cdot r_{m}<0.$$

This condition identifies predefined tool-access directions lying in the admissible hemisphere for the considered table orientation. It does not generate alternative tool-access directions but evaluates whether the predefined direction of each machining feature remains compatible with the candidate workpiece orientation. Surface-pair and orientation combinations satisfying these geometric conditions are subsequently subjected to volumetric collision filtering.

#### 3.4.4. Geometric Collision Filtering

The final geometry-based candidate-generation step evaluates whether sufficient geometric clearance is available adjacent to the selected contact regions. At this stage, the exact position and in-plane orientation of the clamping element are not yet specified. Instead, a pose-independent clearance volume is constructed for each candidate fixturing situation by extruding the selected contact regions along their respective surface normals.

For a candidate fixturing situation *s_h_*, let *V*_clr,*h*_ denote the resulting volumetric clearance region associated with the opposing contact areas. The candidate is retained in workpiece state *X_k_* only if

(25) $$V_{\mathrm{clr},h}\cap V_{k}=\emptyset ,$$

where *V_k_* denotes the occupied workpiece volume in state *X_k_*. Candidates for which the clearance region intersects the occupied workpiece volume are discarded.

The set of geometrically admissible fixturing situations for workpiece state *X_k_* is therefore defined as

(26) $$S_{k}^{\mathrm{fix}}=\left\{s_{h}|V_{\mathrm{clr},h}\cap V_{k}=\emptyset \right\}.$$

Each *s_h_* contains the selected opposing-surface pair, the associated workpiece orientation, and the machining features accessible in that orientation. The resulting set $S_{k}^{\mathrm{fix}}$ constitutes the state-dependent output of the geometry-based fixturing derivation shown in Figure 4.

This check addresses pose-independent geometric clearance; support and collision checks for concrete clamping-element poses follow in Section 3.7. Mechanical feasibility with respect to clamping forces, cutting loads, stiffness, or workpiece deformation remains outside the present model.

### 3.5. Formalisation of the Geometry-Derived Planning Space

Section 3.3 and Section 3.4 provide two complementary components of the planning problem: the mandatory precedence graph *G_P_* and the state-dependent set of geometrically admissible fixturing situations $S_{k}^{\mathrm{fix}}$. These components are combined with the admissible tool alternatives to determine which machining features and resource configurations are selectable in each workpiece state *X_k_*. The resulting representation defines the feasible planning space without requiring all possible machining sequences to be generated explicitly.

#### 3.5.1. Integration of Resources and Fixturing Alternatives

For machining feature *f_i_*, only those fixturing situations in $S_{k}^{\mathrm{fix}}$ that permit its predefined tool-access direction are relevant. Let *F*_acc_(*s_h_*) denote the set of machining features whose predefined tool-access directions are compatible with fixturing situation *s_h_*. The feature-specific set of geometrically admissible fixturing situations in state *X_k_* is therefore defined as

(27) $$S_{i}\left(X_{k}\right)=\left\{s_{h}\in S_{k}^{\mathrm{fix}}|f_{i}\in F_{\mathrm{acc}}\left(s_{h}\right)\right\}.$$

Geometry-based fixturing derivation may yield numerous candidate situations for a single machining feature. To restrict the resource space considered during optimisation, the feature-compatible candidates are first ranked according to the geometric score introduced in Section 3.4.3, and only the highest-ranked *N_S_*_,*max*_ alternatives are retained. This feature-specific preselection is performed before evaluating their availability in an individual workpiece state. Consequently, the retained set *Ŝ_i_*(*X_k_*) consists of those preselected alternatives that are also geometrically admissible in state *X_k_* according to Equation (27). The number of available alternatives may therefore be smaller than *N_S_*_,*max*_ and lower-ranked candidates are not reconsidered when preselected alternatives become unavailable. This truncation restricts the planning space explored by the optimiser without modifying the underlying geometric-admissibility criteria. The reference value of *N_Smax_* is specified in Section 4.1.

(28) $$C_{i}\left(X_{k}\right)=\left\{\left(t,s\right)|t\in T_{i},\;s\in {\hat{S}}_{i}\left(X_{k}\right)\right\}\;$$

Each configuration *c_i_* = (*t*,*s*) ∈ *C_i_*(*X_k_*) therefore combines one admissible cutting tool with one state-dependent fixturing situation.

#### 3.5.2. Dynamic Release in the Operation-Sequence Representation

The precedence graph *G_P_* defines the mandatory geometry-induced ordering constraints, whereas Equation (28) defines the resource configurations available in the current workpiece state. These two information layers are evaluated jointly during sequence construction rather than being converted into an additional static graph of resource-dependent transition edges.

Let Pred(*f_i_*) denote the set of mandatory predecessor features of *f_i_* in *G_P_*. For the set *D_k_* of already completed machining features, the precedence-released feature set is

(29) $$R(D_{k})=\{f_{i}\in F\setminus D_{k}|\mathrm{Pred}(f_{i})\subseteq D_{k}\}$$

A precedence-released feature is selectable only if at least one valid resource configuration is available in the current workpiece state. The selectable feature set is therefore

(30) $$A(X_{k})=\{f_{i}\in R(D_{k})|C_{i}(X_{k})\neq \emptyset \}$$

Equations (29) and (30) jointly determine the operations selectable in a given partial-plan state based on precedence and resource admissibility, respectively, as illustrated in Figure 8 for an exemplary admissible process-plan path.

In Figure 8, *R* denotes the illustrative root node, whereas RF and FF denote roughing and finishing features, respectively. The graph provides a conceptual representation of the sequence space; actual admissibility at each planning step is evaluated dynamically according to Equations (29) and (30).

#### 3.5.3. Feasible Planning Space

A complete process plan consists of an ordering of all machining features and one resource configuration selected for each sequence position. Let

*π* = (*π*_1_, … ,*π_n_*), *n* = ∣*F*∣, (31)

denote a permutation of the machining-feature indices and let *c_k_* denote the resource configuration assigned to the feature executed at sequence position *k*. A complete plan is represented as

(32) $$P=(\pi ,c_{1},\ldots ,c_{n}).$$

A complete plan is feasible only if, at every sequence position *k*,

(33) $$\mathrm{Pred}\;\left(f_{\pi_{k}}\right)\subseteq D_{k-1}$$

Moreover,

(34) $$c_{k}\in C_{\pi_{k}}\;\left(X_{k-1}\right).$$

Let Π*_n_* denote the set of all permutations of the *n* machining-feature indices. The feasible planning space is therefore defined as

(35) $$\Omega =\left\{P=\left(\pi ,c_{1},\ldots ,c_{n}\right)|\pi \in \Pi_{n},\;\forall k\in \left\{1,\ldots ,n\right\}:\mathrm{Pred}\left(f_{\pi_{k}}\right)\subseteq D_{k-1}\wedge c_{k}\in C_{\pi_{k}}\left(X_{k-1}\right)\right\}$$

Thus, Ω contains only complete process plans that satisfy the mandatory precedence constraints and assign an admissible resource configuration to every machining feature in the corresponding workpiece state. The optimiser therefore searches within the feasible planning space rather than generating unrestricted feature permutations and repairing infeasible solutions afterwards.

### 3.6. Search of the Feasible Planning Space Using a Ha-Based Evolving Ant Colony System

With the feasible planning space Ω defined, the optimisation searches for favourable process plans using the EACS proposed by Ha [18]. The term EACS is used throughout for consistency with the source publication; the archived implementation refers to the same Ha-based solver as EACO. The underlying search mechanics follow Ha [18], whereas constraint-compliant Proto-Ant initialisation is used to generate feasible starting solutions that satisfy the precedence, tool, and state-dependent fixturing constraints represented in Ω.

The optimisation algorithm constitutes a downstream search component rather than the principal methodological contribution of the proposed framework. The feasible planning space is defined independently of the selected solver, allowing alternative optimisation methods to be employed provided that they respect the state-dependent admissibility conditions. The Proto-Ant procedure facilitates the efficient generation of admissible initial solutions without modifying the subsequent Ha-based EACS search mechanics [18]. While the initial population may influence the results of a stochastic search under a finite computational budget, no independent improvement in solution quality is claimed for the Proto-Ant procedure. The present study therefore focuses on the construction and exploration of admissible process plans rather than on the comparative performance of optimisation algorithms.

#### 3.6.1. Objective Function

The quality of a complete feasible-process plan *P* is evaluated using the weighted scalar objective:

(36) $$K\left(P\right)=T\left(P\right)+w_{S}N_{S}\left(P\right)+w_{T}N_{T}\left(P\right),$$

where *T*(*P*) denotes the accumulated machining-related abstract cost contribution derived from the machining input data, *N_S_*(*P*) is the number of transitions between different fixturing situations in consecutive operations, and *N_T_*(*P*) is the corresponding number of cutting-tool transitions. The weighting parameters *w_S_* and *w_T_* determine the relative penalties associated with fixturing and tool transitions, respectively. Increasing either parameter shifts the optimisation preference towards plans containing fewer transitions of the corresponding resource type.

All contributions are expressed on the same abstract, dimensionless cost scale. The weighting parameters therefore represent relative planning preferences rather than conversions to monetary cost or physical process time. The parameter values used in the computational evaluation are specified in Section 4.1.

#### 3.6.2. Constraint-Compliant Proto-Ant Initialisation

Unrestricted random initialisation becomes increasingly inefficient as the planning problem grows because arbitrary feature permutations frequently violate mandatory precedence constraints or encounter workpiece states for which no admissible resource configuration is available. Proto-Ants are therefore constructed incrementally from the feasible candidate sets defined in Section 3.5.2.

At each sequence position, the set of precedence-released features is determined according to Equation (29). This set is subsequently restricted according to Equation (30) to features for which at least one admissible resource configuration is available in the current workpiece state. One feature is selected from the resulting candidate set and assigned one configuration from its corresponding resource set *C_i_*(*X_k_*). After execution of the selected feature, the completed-feature set and the workpiece state are updated before the next sequence position is constructed.

No feature that violates the represented precedence or resource constraints is inserted into the partial route. Construction therefore proceeds exclusively through admissible partial plans until a complete feasible initial solution has been generated. Algorithm 3 summarises the construction procedure.
**Algorithm 3.** Constraint-compliant Proto-Ant initialization.constructProtoAnt(F: Set<Feature>, G_P: DirectedGraph, X_0: State): Plan1
plan: List<Operation>:= emptyList()2
D:= ∅3
X:= X_04
**while** |D| < |F| do5│    released:= {f_i ∈ F \ D | Pred(f_i) ⊆ D}6│    admissible:= {f_i ∈ released | C_i(X) ≠ ∅}7│    f:= choose(admissible)8│    c:= choose(C_f(X))9│    plan.pushBack((f, c))10│    D:= D ∪ {f}11│    X:= updateState(X, f)12
**end**13
**return** plan

Within Algorithm 3, *D* denotes the set of already selected machining features, *X* denotes the current workpiece state, released denotes the concrete realisation of *R*(*D*), and admissible denotes the corresponding realisation of *A*(*X*). The function choose(·) denotes the stochastic selection applied during Proto-Ant construction.

#### 3.6.3. Ha-Based EACS Search After Initialisation

After generation of the constraint-compliant initial population, the subsequent search follows the Ha-based EACS implementation used in the underlying planning software [18]. Route construction, preservation and replacement of high-performing ants, and pheromone updating are applied to explore the feasible planning space. Complete process plans are evaluated according to Equation (36), and the admissible solution with the lowest objective value found within the specified computational budget is retained. The procedure is heuristic; no claim of global optimality is made.

The EACS search is governed by parameters controlling the relative influence of accumulated search experience and local heuristic information, as well as the available computational budget. The pheromone exponent *α* controls the influence of pheromone information associated with previously successful decisions, whereas the heuristic exponent *β* determines the influence of the local heuristic desirability term. The pheromone-update parameter *ρ* controls the modification of the pheromone representation during the search. The number of ants per generation *N_A_* and the maximum number of generations *N_G_* jointly determine the principal computational search budget.

Additional parameters control the evolutionary initialisation and variation mechanisms of the implemented solver. *N_P_* denotes the number of constraint-compliant Proto-Ant solutions used to initialise the search and therefore affects the diversity of the starting population. The selected-parent fraction *p*_sel_ specifies the proportion of high-performing solutions retained for the evolutionary update. The mutation probabilities *p_T_*, *p_S_*, and *p*_0_ control modifications of tool assignments, fixturing assignments, and the initial operation, respectively. These parameters govern how the solver explores the previously defined feasible planning space; they do not modify the geometry-derived feasibility conditions. The parameter values used in the computational experiments are reported in Section 4.1.

### 3.7. Pose-Level Instantiation of Selected Fixturing Situations

The optimisation determines which fixturing situation is assigned to each machining operation but does not yet specify the exact pose of the physical clamping element within the selected contact regions. The final methodological step therefore instantiates each selected fixturing situation as a concrete clamping-element pose. This downstream placement refines the selected resource assignment without modifying the operation sequence or reopening the feasible planning space. For a selected pair of opposing fixturing areas *P*_1_ and *P*_2_, the corresponding surface grids are transformed into a common local coordinate system and restricted to grid cells simultaneously available on both contact regions. For each cell (*x*,*y*) of the resulting overlap grid, the local placement score is obtained by combining the suitability values of the two opposing surfaces:

(37) $$S_{\mathrm{ov}}(x,y)=S_{P_{1}}(x,y)+S_{P_{2}}(x,y).$$

Here, $S_{P_{1}}\left(x,y\right)$ and $S_{P_{2}}\left(x,y\right)$ denote the cell-level suitability values contributed by the two opposing fixturing areas at the same projected grid location. The resulting overlap score *S*_ov_(*x*, *y*) therefore favours locations that are geometrically suitable on both contact surfaces.

To reduce the number of exact pose evaluations, candidate fixture centres are first ranked using a circular proxy of the rectangular clamping footprint. The proxy is defined as the circumscribed circle whose diameter equals the diagonal of the rectangular footprint. It is translated over the overlap grid, and the enclosed overlap scores are accumulated for each candidate centre. Candidate centres are then processed in descending order of the resulting proxy score, such that geometrically favourable regions are evaluated first.

At each candidate centre, the actual rectangular clamping footprint is evaluated for a finite set of in-plane orientations:

(38) $$\Theta_{R}=\{\theta_{1},\ldots ,\theta_{N_{R}}\},$$

where *N_R_* controls the angular discretisation of the local placement search. Increasing *N_R_* provides a finer approximation of the continuous orientation space at the expense of additional geometric pose evaluations. For each orientation *θ* ∈ Θ*_R_*, the overlap-grid cells covered by the rotated footprint are identified and their scores accumulated. Poses that are not fully supported by the available contact region or violate the geometric collision constraints are rejected.

For the first candidate centre admitting at least one valid rectangular placement, the admissible orientation with the highest overlap score is retained as the concrete clamping-element pose. The procedure therefore performs a two-stage heuristic search: candidate centres are prioritised using the circular proxy, followed by exact footprint evaluation at the most promising feasible centre. Figure 9 illustrates both stages of the placement procedure.

The complete placement procedure is summarised in Algorithm 4.
**Algorithm 4.** Geometric placement of a standardised clamping element.placeClampingElement(P_a: Area, P_b: Area, footprint: Geometry, orientations: Set<Angle>, X: State): Pose ∪ {⊥}1
G_ov:= overlapGrid(P_a, P_b)2
**for** c ∈ G_ov do3│   proxyScore[c]:= circularProxyScore(c, G_ov, footprint)4
**end**5
candidateCentres:= sortDescending(G_ov, proxyScore)6
**for** c ∈ candidateCentres do7│   validPoses: Set<(Pose, Score)>:= ∅8│   **for** θ ∈ orientations do9│      pose:= place(footprint, c, θ)10│      **if** supported(pose, G_ov) ∧ collisionFree(pose, X) then11│         validPoses:= validPoses ∪ {(pose, poseScore(pose, G_ov))}12│      **end**13│   **end**14│   **if** validPoses ≠ ∅ then15│       **return** argMax(validPoses, score)16│   **end**17
**end**18
**return** ⊥  // no admissible placement

In Algorithm 4, *G*_ov_ denotes the overlap grid, proxyScore[c] the circular-proxy score at candidate centre *c*, and validPoses the set of geometrically admissible poses evaluated at that centre. The procedure returns ⊥ if no admissible placement can be identified. The localisation procedure introduces the additional discretisation parameter *N_R_*, which controls the resolution of the in-plane orientation search. Together with the geometric discretisation, candidate-generation, and EACS parameters introduced in the preceding Sections, it affects the computational effort and resolution of the represented planning problem but does not modify the underlying feasibility definitions.

## 4. Evaluation Methodology

The evaluation addresses the principal stages of the proposed framework separately. The experimental configuration and benchmark first define the investigated planning tasks. Geometric discretisation, precedence derivation, and fixturing generation are then assessed with respect to their corresponding geometric criteria. The stochastic experiments evaluate the search of the resulting feasible planning space, followed by runtime analysis and an expert assessment of the technological plausibility of the generated process plans.

### 4.1. Experimental Configuration and Benchmark

The parameters introduced in Section 3 must be instantiated for the computational evaluation. Table 4 summarises the parameter values used throughout the experiments. These values define the investigated configuration and, unless stated otherwise, are not claimed to represent universally optimal settings. Let *L*_max_ denote the largest edge length of the axis-aligned bounding box (AABB) of the initial workpiece stock. The surface-grid edge length is scaled accordingly as *L_g_* = *L*_max_/100.

The parameter values in Table 4 combine engineering assumptions, heuristic preferences, and settings inherited from the precursor implementation [11]. The minimum projected overlap (*A_min_* = 756 mm^2^) corresponds to the contact footprint of the smallest standardised clamping element (12 × 63 mm), whereas the relative-separation factor (*λ_d_* = 0.5) represents a geometric preselection assumption. Equal suitability weights (*w*_1_ = *w*_2_ = 0.5) balance the two geometric scoring criteria. The setup- and tool-change weights (*w_S_* = 200, *w_T_* = 10) express relative planning preferences rather than measured manufacturing costs. The EACS parameters were retained from the Ha-informed precursor implementation [18] for consistency with the archived benchmark; they are not claimed to be optimal for the investigated planning tasks.

The retention limit *N_S_*_,max_ = 20 was selected conservatively during method development. An exploratory check on the most complex benchmark instance, Part 23, indicated that the resource assignments occurring in the selected solutions originated from the ten highest-ranked fixturing candidates. A limit of 20 was therefore retained to restrict the branching factor while providing an additional margin. Because the underlying exploratory outputs were not preserved, this observation is used solely to motivate the parameter choice and is not considered quantitative validation or evidence that the reduction is lossless for arbitrary planning tasks.

The evaluation uses the 24-part benchmark originally established in [11]. The archived benchmark and run-level data are reused, whereas the statistical analyses and visualisations reported in the present study were generated specifically for this evaluation. The benchmark covers machining tasks with different workpiece dimensions, feature counts, and resource structures, with maximum workpiece dimensions of up to 900 mm and between 3 and 48 machining features. Complete part-level benchmark data are provided in Supplementary Table S1.

For descriptive structuring of the benchmark, the 24 test parts are assigned to four cohorts according to maximum workpiece dimension and machining-feature count, as summarised in Table 5 and Figure 10. Cohorts A and B contain parts with maximum dimensions of up to 400 mm, whereas Cohorts C and D contain larger parts. Cohorts A and C comprise parts with 3–14 machining features, whereas Cohorts B and D comprise parts with 15–50 features.

All 24 parts are used for the quantitative evaluation. Parts 2, 10, 13, and 23 are additionally examined as illustrative cases in Section 5.4, with one case drawn from each cohort.

An additional exploratory one-factor-at-a-time sensitivity analysis was performed on Parts 2, 10, 13, and 23, representing the four benchmark cohorts. The investigation considered three parameter groups: surface-grid resolution (*Lmax*/75, *Lmax*/100, and *Lmax*/125), geometric fixturing-suitability weights ((0.25, 0.75), (0.50, 0.50), and (0.75, 0.25)), and the maximum number of retained fixturing alternatives (*N_S_*_,*max*_ = 10, 20, and 30). Geometric candidate availability was assessed for fixed feature–state combinations derived from feasible reference sequences. Variations in the candidate limit were determined by applying the original ranking and truncation procedure to the recorded candidate pools. Additionally, five independent EACS runs were conducted for each candidate-limit configuration of Parts 10 and 13 using identical sets of random seeds and a fixed search budget of 100 ants per generation and 100 generations. The octree depth and objective-function weights were kept constant. The influence of extreme objective-criterion weighting was previously investigated in [11]. The resulting sensitivity analysis distinguishes changes in the geometry-derived planning space from the outcomes of its subsequent stochastic exploration.

All computational experiments, including the original benchmark and the additional sensitivity analysis, were performed on an HP Z840 workstation (HP Inc., Palo Alto, CA, USA) equipped with two Intel Xeon E5-2667 v4 processors operating at 3.20 GHz (16 physical cores and 32 threads in total) and 128 GB of ECC DDR4 memory at 2400 MHz. The optimisation algorithm was executed as a single-threaded implementation. Reported runtimes refer to computation only and exclude file loading, visualisation, and result export.

### 4.2. Verification of Geometry-Derived Planning Information

#### 4.2.1. Geometric Accuracy and Precedence

Geometric accuracy is assessed for the volumetric workpiece representation, the projected surface grid, and the centre-ray accessibility analysis. Here, the discretisation bounds quantify the maximum spatial extent of the respective discrete cells and thus provide conservative resolution limits of the geometric representations. For an octree root-volume edge length *L_v_* and maximum depth *T*, the bound corresponding to the space diagonal of the finest volumetric cell is

(39) $$\delta_{\mathrm{vox}}=\frac{\sqrt{3}L_{v}}{2^{T}}.$$

For the projected surface grid, the corresponding in-plane discretisation bound is given by the diagonal of a grid cell with edge length *L_g_*:

(40) $$\delta_{\mathrm{grid}}=\sqrt{2}L_{g}.$$

The centre-ray accessibility analysis introduces an additional sampling limitation. For ray length l, octree root-volume edge length *L_v_*, maximum depth *T* and angular difference Δ*θ* between the access direction and the relevant grid orientation, the lateral sampling deviation is conservatively approximated by

(41) $$\varepsilon_{\mathrm{FN}}≲\frac{\sqrt{3}}{2}\frac{L_{v}}{2^{T}}+l\tan\left(\Delta \theta \right).$$

The first term in Equation (41) corresponds to half the space diagonal of the finest voxel and bounds the displacement between its centre and any point within the cell. The second term approximates the lateral deviation associated with the angular offset over the considered ray length. Their sum provides a conservative geometric estimate under the assumption that both offsets act in the same direction. It does not represent an empirical false-negative probability or guarantee the detection of arbitrarily narrow obstructions. Figure 11 illustrates the underlying discretisation and sampling effects.

#### 4.2.2. Verification of Geometry-Derived Fixturing Candidates

Geometry-derived fixturing candidates are subjected to an ex-post verification against geometric acceptance criteria stated independently of the candidate-generation procedure. Candidate surface pairs are generated using the normal-opposition, projected-overlap, and relative-separation criteria defined in Section 3.4.3. The retained candidates are subsequently evaluated against ex-post geometric acceptance criteria comprising a maximum planarity deviation of 0.1 mm, a minimum usable contact area of 756 mm^2^, sufficient projected overlap, absence of volumetric collision, and the angular acceptance criterion associated with tolerance class *c* according to ISO 2768-1 [54].

The minimum contact-area threshold corresponds to the 12 mm × 63 mm footprint of the smallest standardised clamping element represented in the fixture database. The ISO-based angular criterion is used exclusively for verification and is not part of the internal normal-opposition rule used during candidate generation. Similarly, the relative-separation criterion introduced in Section 3.4.3 remains a heuristic preselection criterion and is not interpreted as evidence of mechanical fixture stability. The comparison therefore assesses consistency between the implemented candidate-generation procedure and independently stated geometric acceptance criteria rather than the mechanical quality of the resulting fixturing situations.

The implementation of the fixturing suitability map is additionally verified using the isolated weighting configurations *w*_1_ = 1, *w*_2_ = 0 and *w*_1_ = 0, *w*_2_ = 1. The first configuration isolates the boundary-distance criterion, whereas the second isolates the volume-centroid criterion. The resulting suitability fields are examined to verify that increasing boundary distance and decreasing distance to the associated material-removal-volume centroid influence the cell-level suitability scores in the intended directions. This analysis constitutes an implementation check of the geometric scoring model and is not interpreted as validation of mechanical fixture stability.

#### 4.2.3. Verification of Clamping-Element Placement

The clamping-element placement procedure introduced in Section 3.7 is evaluated separately against an exhaustive model-internal reference. Parts 6, 7, 13, and 24 are selected for this analysis to cover different workpiece dimensions, geometric conditions, and fixturing situations.

For each investigated fixturing situation, all admissible clamping-element poses are exhaustively enumerated over the implemented fixture types, candidate positions, and discrete in-plane orientations. Each enumerated pose is evaluated using the same geometric contact, coverage, suitability, and collision criteria as the proposed placement procedure. The exhaustive search therefore provides the best attainable placement according to the defined geometric pose model.

The pose returned by the proposed two-stage placement procedure is compared with this exhaustive reference using three quantities: its rank among all admissible poses, the total number of admissible poses identified by exhaustive enumeration, and the relative deviation of its placement score from the exhaustive-reference optimum. The relative deviation is defined as

(42) $$\Delta_{\mathrm{rel}}=\frac{S_{\mathrm{ref}}-S_{\mathrm{prop}}}{S_{\mathrm{ref}}}\cdot 100\%,$$

where *S*_ref_ denotes the highest placement score obtained by exhaustive enumeration and *S*_prop_ denotes the score of the pose returned by the proposed placement procedure.

This comparison assesses how closely the heuristic two-stage search approaches the best solution available within the defined discrete geometric pose space. Because the exhaustive reference is based on the same geometric model as the proposed placement procedure, it constitutes a model-internal verification rather than an independent physical validation. In particular, the comparison does not assess clamping-force transmission, fixture stiffness, workpiece deformation, process forces, or machining performance.

### 4.3. Statistical Evaluation of Planning-Space Search

The stochastic evaluation uses the archived run-level objective values for all 24 benchmark parts. For each part, 58 independent EACS optimisation runs and 1000 random admissible reference solutions are available, resulting in 1392 EACS solutions and 24,000 random-reference solutions across the complete benchmark.

At each construction step, a machining feature is uniformly sampled from the currently admissible candidates. An admissible tool and fixturing situation are subsequently selected uniformly from their respective candidate sets. The workpiece state is updated accordingly, and only complete admissible plans are retained. Thus, sampling is uniform at the decision level, but not over the complete feasible planning space Ω. A new pseudorandom seed was generated for each batch of runs; the original seeds were not archived.

The comparison is intended to demonstrate that the constructed planning space can be explored by an established guided search method and that admissible process plans with lower objective values can be obtained than through unguided sampling. The EACS algorithm was adopted from Ha [18], who previously investigated its optimisation performance for integrated process-planning and scheduling problems. These previous findings do not directly establish the performance of EACS for the geometry-based planning problems considered here. Accordingly, random admissible sampling serves as an unguided, feasibility-preserving reference rather than as a competitive optimisation benchmark or an assessment of proximity to the global optimum.

For benchmark part *i*, the distributional separation between EACS and random admissible sampling is quantified using the empirical probability of superiority:

(43) $$A_{i}=\frac{1}{N_{E}N_{R}}\sum_{r=1}^{N_{E}}\sum_{s=1}^{N_{R}}\left[I\left(K_{i,r}^{E}<K_{i,s}^{R}\right)+\frac{1}{2}I\left(K_{i,r}^{E}=K_{i,s}^{R}\right)\right],$$

where *N_E_* and *N_R_* denote the numbers of EACS and random-reference solutions, respectively; $K_{i,r}^{E}$ and $K_{i,s}^{R}$ denote the corresponding objective values for benchmark part *i*; and I(·) denotes the indicator function, which equals one if the enclosed condition is true and zero otherwise. Accordingly, *A_i_* = 0.5 indicates no systematic distributional advantage, whereas values approaching 1 indicate increasing separation in favour of EACS. For the across-part statistical analysis, the benchmark part is used as the unit of comparison to avoid treating repeated optimisation runs as independent observations. The 24 parts constitute a fixed benchmark rather than a representative random sample of industrial machining tasks. For each part, the mean EACS objective value ${\bar{K}}_{i}^{E}$ and the mean random-reference objective value ${\bar{K}}_{i}^{R}$ are calculated. The direction of the part-level difference

(44) $$\Delta_{i}={\bar{K}}_{i}^{R}-{\bar{K}}_{i}^{E}$$

is then evaluated across the benchmark. Positive values of Δ*_i_* indicate a lower mean objective value for EACS.

To test whether the direction of the part-level differences is consistent across the benchmark, an exact two-sided sign test is applied. Under the null hypothesis, positive and negative non-zero differences are assumed to be equally likely. Parts with an exact zero difference are excluded from the test. The exact *p*-value and the proportion of parts with Δ*_i_* > 0 are reported together with the corresponding exact 95% binomial confidence interval.

The probability-of-superiority values *A_i_* provide a distribution-level measure for each individual benchmark part, whereas the sign test evaluates whether the direction of the EACS advantage is consistent across the 24-part benchmark. The two analyses are therefore complementary: the former quantifies within-part separation of the run-level objective distributions, while the latter summarises directional consistency across the 24 benchmark parts. The sign-test results and binomial confidence interval are interpreted under their statistical assumptions and do not support population-level generalisation beyond the investigated benchmark.

### 4.4. Expert-Based Technological Plausibility Assessment

A structured qualitative in-person assessment was conducted with four experienced CAM practitioners. Two participants were affiliated with TUD Dresden University of Technology and two with H+E Produktentwicklung GmbH. Participants were purposively selected from the project-related university and industrial environment based on their practical experience in CAM planning and machining. Their professional backgrounds covered CNC team leadership, process preparation, workshop management, and CAM planning, with 12–30 years of experience in three-axis and five-axis machining environments. Table 6 summarises the participant characteristics.

The experts assessed generated process plans for Parts 6, 7, 13 and 24 using the interactive CAD/CAM-like demonstrator shown in Figure 12. For each operation, the demonstrator provided the current workpiece state, selected fixturing situation, clamping-element positions and tool information. Before the assessment, the system boundary was explained, including the restriction to indexed 3 + 2-axis milling, standardised prismatic clamping elements, predefined tool-access directions, and geometric rather than force-based fixturing analysis.

The assessment addressed three aspects: technological realisability of the generated process plan and displayed tool-access directions, practical realisability of the proposed fixturing situations and clamping-element positions, and usefulness of the approach for supporting CAM planning. The first two aspects were assessed separately for each of the four test parts using a categorical judgement supplemented by free-text comments. Usefulness was assessed once overall, again with an explanatory comment.

Categorical responses are evaluated descriptively, while the free-text comments are analysed for recurring observations concerning operation ordering, fixturing, process stability and practical applicability. The sample is treated as a purposive expert assessment rather than as a statistically representative survey.

## 5. Results

### 5.1. Geometric Accuracy and Precedence-Graph Consistency

The discretisation analysis quantifies the scale dependence of the volumetric representation. For a 400 mm characteristic root dimension, the conservative geometric bound decreases from approximately 10.83 mm at T = 6 to 0.68 mm at T = 10. For a 1000 mm characteristic dimension, the corresponding values decrease from approximately 27.06 mm to 1.69 mm. Thus, the same octree depth does not provide a scale-independent geometric resolution; finer discretisation is required as the represented workpiece volume increases.

A corresponding relationship is obtained for the projected surface grid used in the fixturing analysis. Grid-cell edge lengths of 2, 1, 0.5 and 0.25 mm result in conservative in-plane bounds of approximately 2.8, 1.4, 0.7 and 0.35 mm, respectively. These results emphasise that both volumetric and surface-based discretisation parameters must be selected with respect to workpiece scale and the geometric accuracy required for planning.

The centre-ray accessibility formulation introduces an additional sampling limitation. Because one ray is emitted from each start-voxel centre, sufficiently narrow obstructions may remain undetected when their projection lies between neighbouring centre rays. The analytical relationship derived in Section 4.2.1 therefore characterises a conservative false-negative detection region rather than an empirical false-negative probability; no global detection-error rate is inferred from this bound.

Functional verification of the precedence-graph construction was performed for all 24 benchmark parts. For every part, the generated graph contained the complete predefined feature set, exhibited no inconsistencies with the geometry-induced dependencies identified during functional verification, and was reproduced deterministically from unchanged input data. These checks are interpreted as functional consistency verification rather than validation against an independently established precedence ground truth.

### 5.2. Fixturing Rule Consistency and Placement Quality

#### 5.2.1. Geometry-Derived Fixturing Candidates

Across the complete test basis, 573 fixturing situations retained after the geometric preselection stage were checked against the ex-post geometric verification criteria defined in Section 4.2.2. The number of evaluated combinations ranged from four for Part 8 to 93 for Part 23. No violation of the planarity, minimum-area, angular-opposition, projected-overlap, or volumetric-collision criteria was observed among the retained combinations. This result supports consistency between the internal candidate-generation logic and the independently stated geometric acceptance limits. It does not validate the intuitive separation heuristic as a mechanical stability criterion.

The isolated weighting tests of the suitability map behaved in the intended direction. With *w*_1_ = 1 and *w*_2_ = 0, high scores shifted towards cells further from area boundaries. With *w*_1_ = 0 and *w*_2_ = 1, the score distribution shifted towards regions closer to the centre of mass of the removal volume. These tests verify implementation of the geometric scoring rule rather than mechanical optimality; the numerical response is retained as supporting implementation evidence rather than as a separate main-text figure.

#### 5.2.2. Clamping-Element Placement Versus Exhaustive Reference

The downstream placement module was compared with the exhaustive model-internal reference search for Parts 6, 7, 13 and 24. The sliding-window localisation identified the highest-rated contact region according to the implemented geometric objective, after which the selected discrete clamping-element pose was compared with all admissible poses generated by exhaustive enumeration. For Parts 6, 7 and 24, the mean relative deviation from the exhaustive-reference optimum remained below 1%. Part 13 showed a higher mean deviation of 6.39%, indicating a larger discrepancy between the heuristic placement and the exhaustive model-internal reference for this part. The detailed placement results are summarised in Table 7.

Across the four representatives, the selected poses ranked on average within approximately the best 2.8% of the enumerated admissible alternatives. These results indicate that the proposed search identifies high-ranking poses within the implemented geometric placement model. As both the heuristic and exhaustive reference use the same geometric criteria, the comparison constitutes model-internal verification and does not establish mechanical stability, clamping-force adequacy, or machining performance.

### 5.3. Planning-Space Search Performance Across the 24-Part Test Basis

Recalculation from the archived run-level objective values shows pronounced distributional separation for all 24 test parts. For 23 parts, every observed EACS objective value was lower than every corresponding random-reference value, yielding *A_i_* = 1.0. For Part 2, none of the 1000 random-reference solutions outperformed the EACS result, whereas 32 attained the same minimum objective value. Accounting for ties gives *A*_2_ = 0.9840.

The mean of the 24 part-level probability-of-superiority values is 0.99933. The near-saturated effect size is therefore reported together with the number of benchmark parts exhibiting complete observed distributional separation rather than interpreted as an exact population probability. An observed probability of superiority of *A_i_* = 1 indicates complete separation of the sampled objective-value distributions, but does not establish global optimality. The pronounced separation should be considered in light of the objective-function weighting (*w_S_* = 200, *w_T_* = 10) and the unguided resource selection of the random reference. For Part 23, EACS objective values ranged from 512 to 1135, compared with 7452 to 11,338 for random sampling. These differences are consistent with guided search favouring low-cost operation and resource assignments. However, the available data do not permit the individual contributions of objective weighting, benchmark characteristics, search behaviour, and Proto-Ant initialisation to be isolated. The latter facilitates feasible initialisation but does not modify the subsequent EACS search mechanics.

The mean EACS objective value was lower than the corresponding random-reference mean for all 24 test parts. For the observed 24/24 directional outcome, the exact two-sided sign-test probability is

(45) $$p=2{\left(\frac{1}{2}\right)}^{24}=1.19\times 10^{-7}$$

The observed directional success proportion is 24/24 = 1.00, with an exact 95 % binomial confidence interval of approximately 0.858 to 1.000. These results demonstrate consistent directional differences within the investigated benchmark. However, as the test parts were not randomly sampled from a defined population of industrial machining tasks, the statistical results do not establish the generality of this effect beyond the benchmark. The resulting across-part statistics are summarised in Table 8.

### 5.4. Detailed Analysis of Selected Benchmark Parts

The across-part analysis establishes the consistency of the optimisation effect across the complete benchmark but does not illustrate how geometric scale and combinatorial problem size manifest at the level of individual instances. Four benchmark parts, one from each descriptive cohort introduced in Section 4.1, are therefore examined as illustrative cases to provide a more detailed view of instances with differing geometric and planning characteristics. Their characteristics are summarised in Table 9.

Here, *n* = ∣*F*∣ denotes the number of machining features, RF and FF the numbers of roughing and finishing features, respectively, and TADs the number of distinct predefined tool-access directions. The value *n*! characterises only the unconstrained operation-order component and must not be interpreted as the cardinality of the complete feasible planning space Ω.

Figure 13 shows the corresponding empirical objective-value distributions obtained from EACS optimisation and random admissible sampling.

Part 2 contains only three machining features and therefore six unconstrained feature permutations. None of the 1000 random-reference samples attained an objective value below the EACS result, although 32 attained the same minimum. The resulting *A*_2_ = 0.9840 therefore reflects equivalent solutions within the comparatively small admissible planning space.

Parts 10 and 13 illustrate that physical workpiece size and combinatorial sequencing complexity are distinct dimensions. Part 13 is geometrically large but contains only 14 machining features, whereas the substantially smaller Part 10 contains 31 features. For both parts, all observed EACS objective values are lower than the corresponding random-reference values, yielding *A_i_* = 1.0000. Part 23, with 48 machining features and 48! = 1.24 × 10^61^ unconstrained orders, shows the same complete observed separation. These cases illustrate the advantage of guided search over the specified random admissible reference for the investigated planning instances, but they do not establish a general advantage over alternative optimisation algorithms or an asymptotic relationship between feature count and search performance.

Overall, the representative cases show that physical workpiece size alone is not a sufficient indicator of planning complexity. This motivates the following analysis of computational effort across the benchmark.

### 5.5. Runtime and Scalability

The geometry-based preprocessing modules account for comparatively small fractions of total computation time in the reported representative cases. Precedence derivation and potential fixturing-area generation remain modest, and the downstream geometric fixture-placement step is within the order of seconds. The EACS-based operation-sequence optimisation dominates the total runtime. The module-level comparison is shown in Figure 14a.

For the representative cases used in the module-level comparison, operation-sequence optimisation required approximately 100 to 320 s per EACS run. Across the broader test basis, planning tasks with fewer than approximately 15 features remained within the order of seconds to a few minutes. Runtime increased strongly for feature-rich instances and reached approximately 151 to 160 min for the 46- and 48-feature cases. The empirical relationship between feature count and mean runtime is shown in Figure 14b.

Within the investigated 24-part benchmark, runtime increased disproportionately for instances with higher feature counts. However, feature count was not varied independently of precedence-graph density, the number of tool and fixturing alternatives, or geometric complexity. The observed relationship is therefore descriptive and does not establish a general computational complexity law or isolate the influence of feature count. Given the substantial runtimes observed for feature-rich instances, the current implementation should primarily be regarded as an offline planning method.

### 5.6. Parameter Sensitivity

An exploratory sensitivity analysis was conducted to investigate the influence of selected geometric parameters and the retained-fixturing limit on the represented planning space and the subsequent optimisation outcomes. The analysis distinguishes between changes in the available resource alternatives and the performance of the stochastic search, which additionally depends on the prescribed objective-function weights and computational budget.

#### 5.6.1. Influence of the Retained-Fixturing Limit

The integrated consideration of operation sequencing, tool selection, and fixturing decisions introduces a combinatorial planning problem in which additional resource alternatives increase the number of possible decisions during optimisation. The retention limit therefore serves as a computational mechanism for restricting the represented search space. Table 10 summarises the accumulated numbers of state-admissible fixturing candidates for the investigated feature–state combinations.

The reference limit of 20 reduced the available candidate counts by 12.2%, 25.1%, and 26.3% for Parts 10, 13, and 23, respectively. A further reduction to 10 would exclude an additional 35.4%, 45.0%, and 56.7% of the candidates retained at the reference setting. Conversely, increasing the limit from 20 to 30 yielded comparatively smaller gains in candidate availability, particularly for Part 23, where only 112 additional candidates were retained across the investigated states.

These findings support the rationale for selecting 20 as a predefined compromise between reducing the combinatorial search space and preserving a broader range of geometrically suitable fixturing alternatives. This distinction is relevant because the geometric suitability ranking of an individual configuration does not necessarily reflect its contribution to a complete process plan.

#### 5.6.2. Influence on Optimisation Outcomes

The effect of the retention limit on the subsequent EACS search was investigated for Parts 10 and 13 using five independent runs per configuration with an identical nominal search budget. For Part 10, the median objective values were 583.95, 967.17, and 884.30 for limits of 10, 20, and 30, respectively. The corresponding values for Part 13 were 1386.52, 1546.99, and 1118.83.

The non-monotonic results demonstrate that increasing the number of available candidates does not necessarily improve the solutions found under a fixed stochastic search budget. While a larger retained candidate set provides additional planning alternatives, it also changes the search space explored by the optimiser. The computational effect was evident for Part 10, where the median optimisation runtime increased from 16.49 s to 31.34 s when the limit was raised from 20 to 30. Furthermore, for Part 13, alternatives ranked beyond position 20 were selected in all five runs with a retention limit of 30, confirming that configurations excluded by the reference truncation can contribute to the resulting plans.

#### 5.6.3. Influence of Geometric Parameters

The sensitivity analysis also revealed changes in candidate availability resulting from variations in the geometric suitability weights and surface-grid resolution. For Part 23, changing the suitability weights from (0.50, 0.50) to (0.75, 0.25) reduced the accumulated number of retained candidates from 2591 to 1251. Refining the surface grid from *Lmax*/100 to *Lmax*/125 reduced the corresponding number to 1868, while the measured geometric computation time increased from 39.9 s to 53.2 s. These observations demonstrate that the geometric representation and candidate-ranking parameters influence the resulting resource space. The runtime differences are descriptive because only individual geometric executions were considered.

Overall, the results demonstrate that the investigated parameters influence the size and composition of the represented planning space, whereas the quality of the solutions obtained within this space additionally depends on the objective-function weighting and the available search budget. The retention limit of 20 should therefore be understood as a predefined computational compromise rather than a universally optimal or lossless setting. The sensitivity analysis quantifies the effects of this choice for the investigated benchmark instances and identifies the limitations associated with restricting the available fixturing alternatives.

### 5.7. Technological Plausibility from Expert Assessment

All four experts assessed the generated process plans for Parts 6, 7, 13 and 24 as technologically plausible within the defined system boundary, resulting in 16 positive part-level assessments. The proposed fixturing situations and clamping-element positions likewise received 16 positive assessments regarding their practical plausibility.

The categorical judgements were accompanied by differentiated qualitative comments. Experts recommended completing associated roughing operations before finishing operations where possible and reducing unnecessary changes in machining plane or workpiece side. The feature structure of Part 6 was considered unusual by one participant. For Part 7, one expert identified an additional setup compared with a manually refined plan while still considering the generated sequence manufacturable.

The fixturing-related comments further identified limitations of the geometric representation. For Part 7, clamping-element size and required clamping force were raised as practical concerns. For Part 13, inclined clamping surfaces were associated with increased setup effort and a possible need for additional support against tipping. For Part 24, deformation caused by opposing clamping forces was identified as a potential issue. Mechanical forces, workpiece support and thermal influences were repeatedly mentioned as aspects not represented by the current geometric model.

All four participants assessed the approach as potentially useful for CAM-planning support. Suggested application areas included process preparation, CAM planning, quotation preparation and manufacturing-strategy development. Given the purposive selection of experts and the restriction to four benchmark parts, the assessment provides qualitative evidence of technological plausibility but is not statistically representative and does not constitute independent validation of the complete 24-part benchmark. Furthermore, the assessment does not establish mechanical fixture stability under actual machining loads or constitute a systematic comparison with manually optimised expert plans.

## 6. Summary and Conclusions

This study formalised automated process planning for 3 + 2-axis milling as a solver-independent, state-dependent feasible planning space. The framework integrates geometry-induced precedence, evolving workpiece states, state-dependent fixturing alternatives, and admissible tool and tool-access information before optimisation.

The evaluation demonstrates the functional consistency of the geometry-to-planning transformation across the investigated 24-part benchmark. All generated precedence graphs contained the complete feature sets and were consistent with the expected geometry-induced dependencies. Furthermore, all 573 retained fixturing situations satisfied the specified geometric admissibility criteria. For the four parts evaluated by exhaustive pose enumeration, the downstream clamping-element placement remained close to the best solution under the implemented geometric objective, with mean deviations below 1% for three parts and 6.39% for the remaining part. These results indicate that the proposed geometric representation can consistently translate evolving workpiece states into planning constraints and resource alternatives.

The optimisation experiments further show that the resulting planning space contains exploitable structure. EACS achieved lower mean objective values than unguided admissible sampling for all 24 benchmark parts, with complete observed distributional separation for 23 parts and a probability of superiority of *A*_2_ = 0.9840 for the remaining small instance. The comparison does not establish superiority of EACS over alternative optimisation algorithms; rather, it demonstrates that guided search can effectively exploit the automatically constructed feasible planning space. At the same time, the strong increase in runtime for feature-rich instances shows that search complexity, rather than geometric preprocessing, currently constitutes the principal computational bottleneck.

The additional sensitivity analysis showed that geometric parameterisation and candidate-space truncation affect the number and composition of the available fixturing alternatives. The non-monotonic changes in the obtained objective values further demonstrate that expanding the represented planning space does not necessarily improve the solutions found under a fixed stochastic search budget. These results are conditional on the prescribed objective-function weighting. The predefined retention limit of 20 therefore represents a computational compromise between restricting combinatorial complexity and preserving fixturing alternatives, rather than a universally optimal setting.

The expert assessment provides an additional practical perspective. All four participating CAM practitioners assessed the presented process plans as technologically plausible and the associated fixturing situations and clamping-element placements as practically plausible within the defined system boundary. Their comments nevertheless identified aspects that are not represented by the current geometric abstraction, particularly clamping forces, workpiece support, deformation and practical setup considerations. The expert evaluation therefore supports the technological plausibility of the generated plans but should not be interpreted as physical validation or as a comparison with manually optimised expert plans.

Taken together, the results lead to two main conclusions. First, the evolving workpiece state can serve as a common representation linking geometry, operation sequencing and fixturing. Machining decisions modify the workpiece state, and the resulting state in turn determines which subsequent operations and resource configurations are admissible. The planning problem is therefore not adequately represented by a static set of operations and precedence relations alone; admissibility changes as a consequence of preceding manufacturing decisions. Second, the explicit representation of these dependencies allows the construction of the feasible planning space to be separated from the optimisation method used to search it. The principal methodological contribution is therefore the transformation of geometric and manufacturing information into a state-dependent feasible planning space rather than the EACS optimisation itself. This separation provides a basis for applying alternative search and optimisation methods to the same explicitly constructed planning problem.

The conclusions are subject to several limitations resulting from the adopted modelling abstraction. Geometric accuracy depends on voxel and surface-grid resolution, and the centre-ray accessibility formulation may fail to detect sufficiently narrow projected obstructions. The method assumes predefined machining features and one fixed TAD per feature; consequently, the derived precedence and fixturing relations are conditional on these inputs. The fixturing candidate space is additionally reduced through ranking and truncation. The machine tool is represented only at an abstract geometric level and does not include complete machine kinematics, workspace constraints, spindle-head geometry, tool-holder collisions, or detailed clamp accessibility. Most importantly, the fixturing model establishes geometric admissibility rather than mechanical stability; cutting and clamping forces, stiffness, local contact pressure, deformation and thermal effects are not considered. The objective function likewise represents relative planning preferences on an abstract dimensionless scale rather than physical process time or monetary manufacturing cost. Finally, the original 24-part benchmark and its archived run-level data originate from the precursor study. Although the additional sensitivity experiments extend the evaluation, they are based on selected parts from the same benchmark and therefore do not constitute an independent external validation set.

Future work should extend the state-dependent planning representation towards mechanically informed fixturing, alternative feature interpretations and tool-access directions, more detailed machine constraints, adaptive geometric resolution, and adaptive strategies for retaining fixturing alternatives. The observed runtime increase also motivates more efficient search strategies for feature-rich planning tasks. Independent validation using additional industrial components, expert-generated reference plans, and physical machining experiments would further establish the applicability of the framework beyond the investigated benchmark.

## Figures and Tables

**Figure 1:**
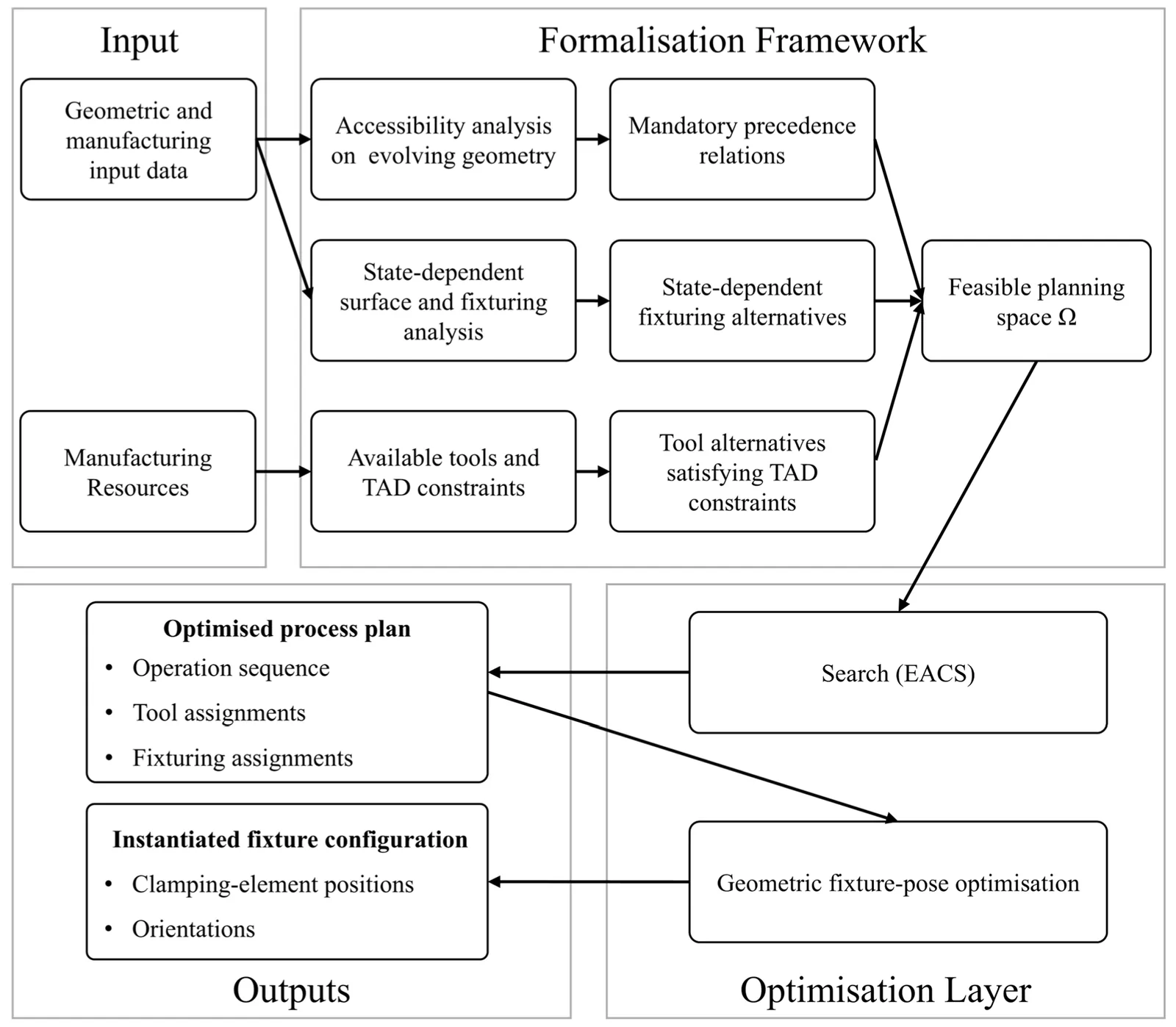
Planning-space formalisation and optimisation workflow.

**Figure 2:**
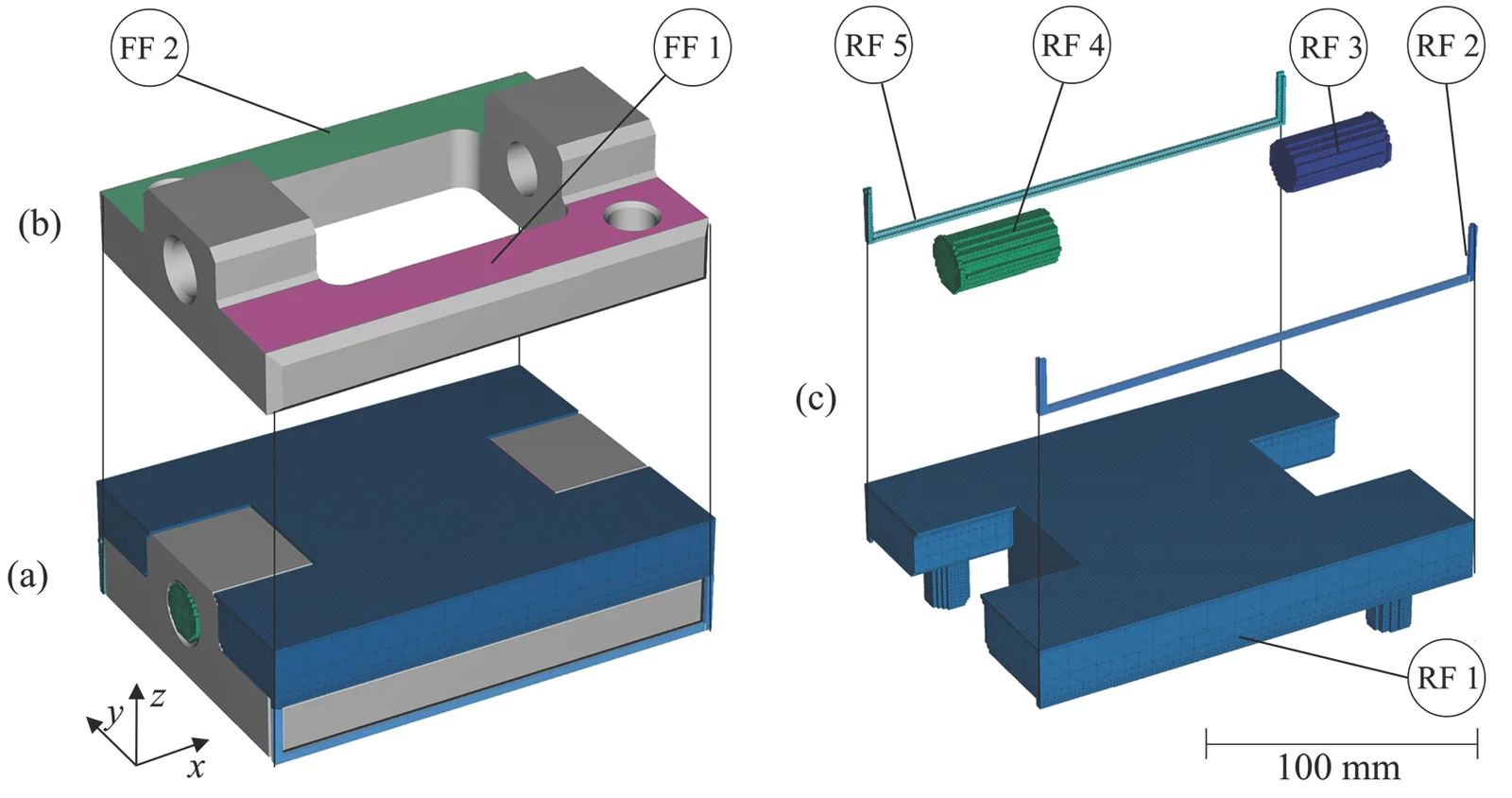
Manufacturing-task geometry: (**a**) volumetric material representation, (**b**) finished-part surface model with finishing features (FFs), and (**c**) roughing features (RFs) as material-removal volumes.

**Figure 3:**
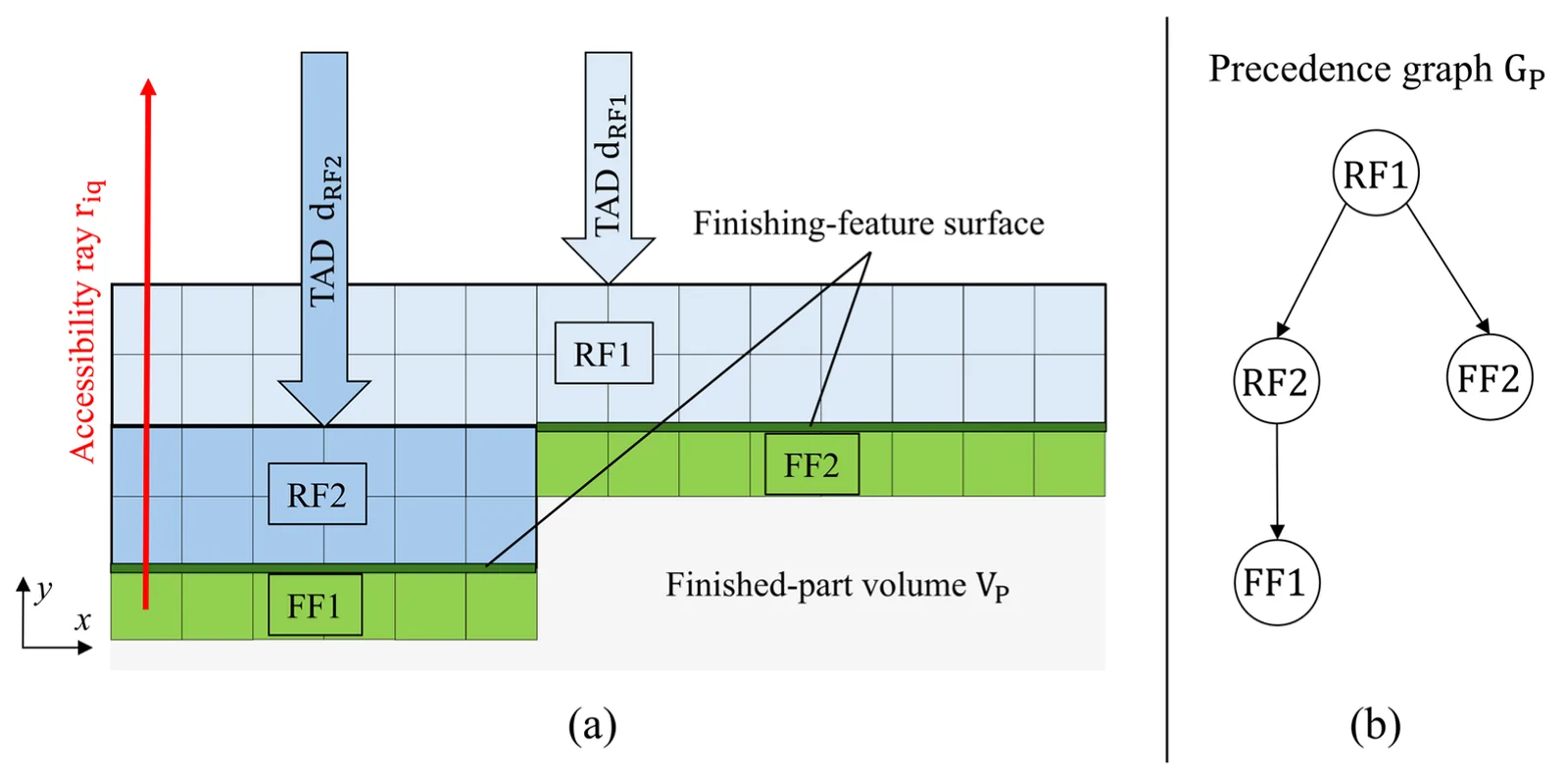
Derivation of geometry-induced machining precedence constraints: (**a**) voxel-based accessibility analysis with accessibility rays cast opposite to the predefined tool-access directions and (**b**) resulting directed precedence graph.

**Figure 4:**
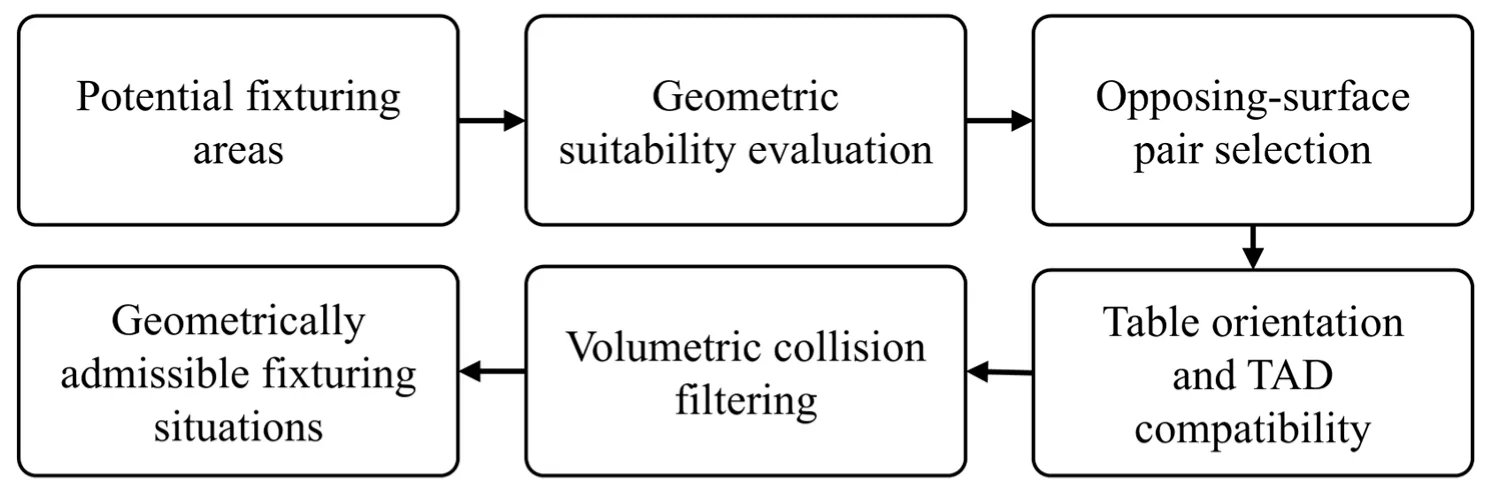
Geometry-based derivation of admissible fixturing situations.

**Figure 5:**
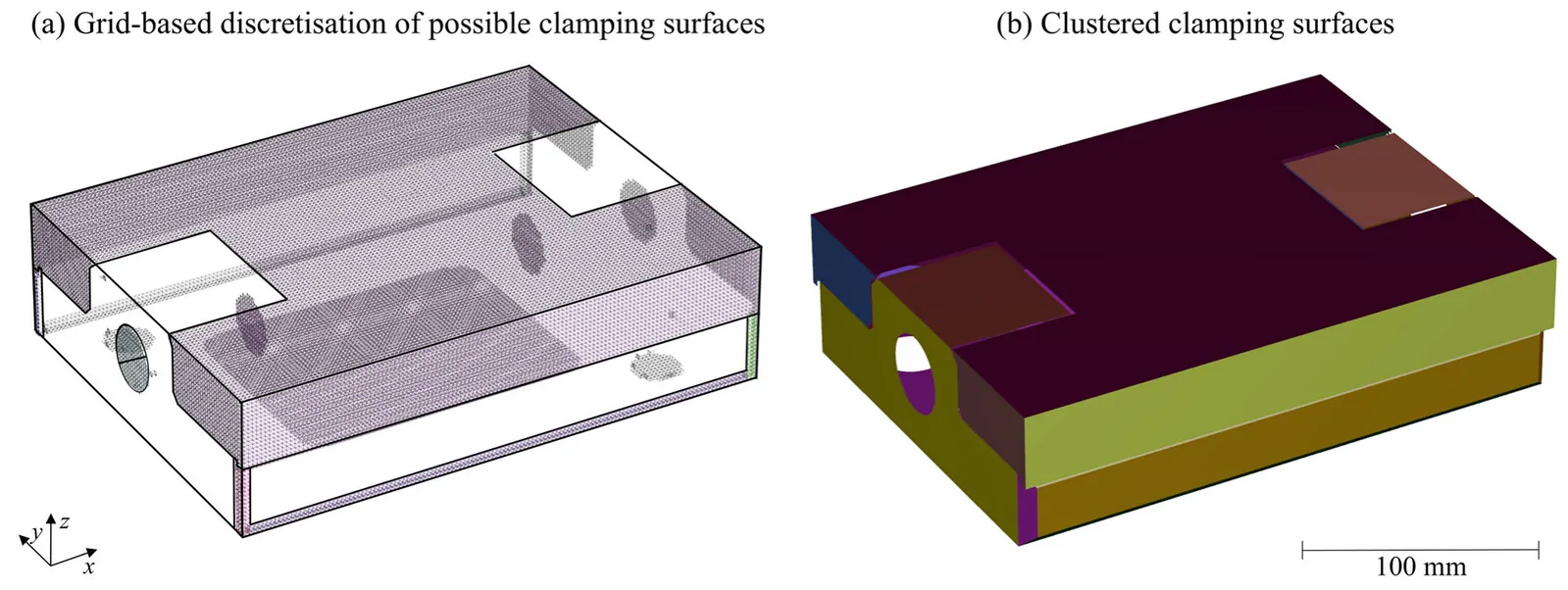
Grid-based surface representation and resulting clustered surface segments. Adapted from [11].

**Figure 6:**
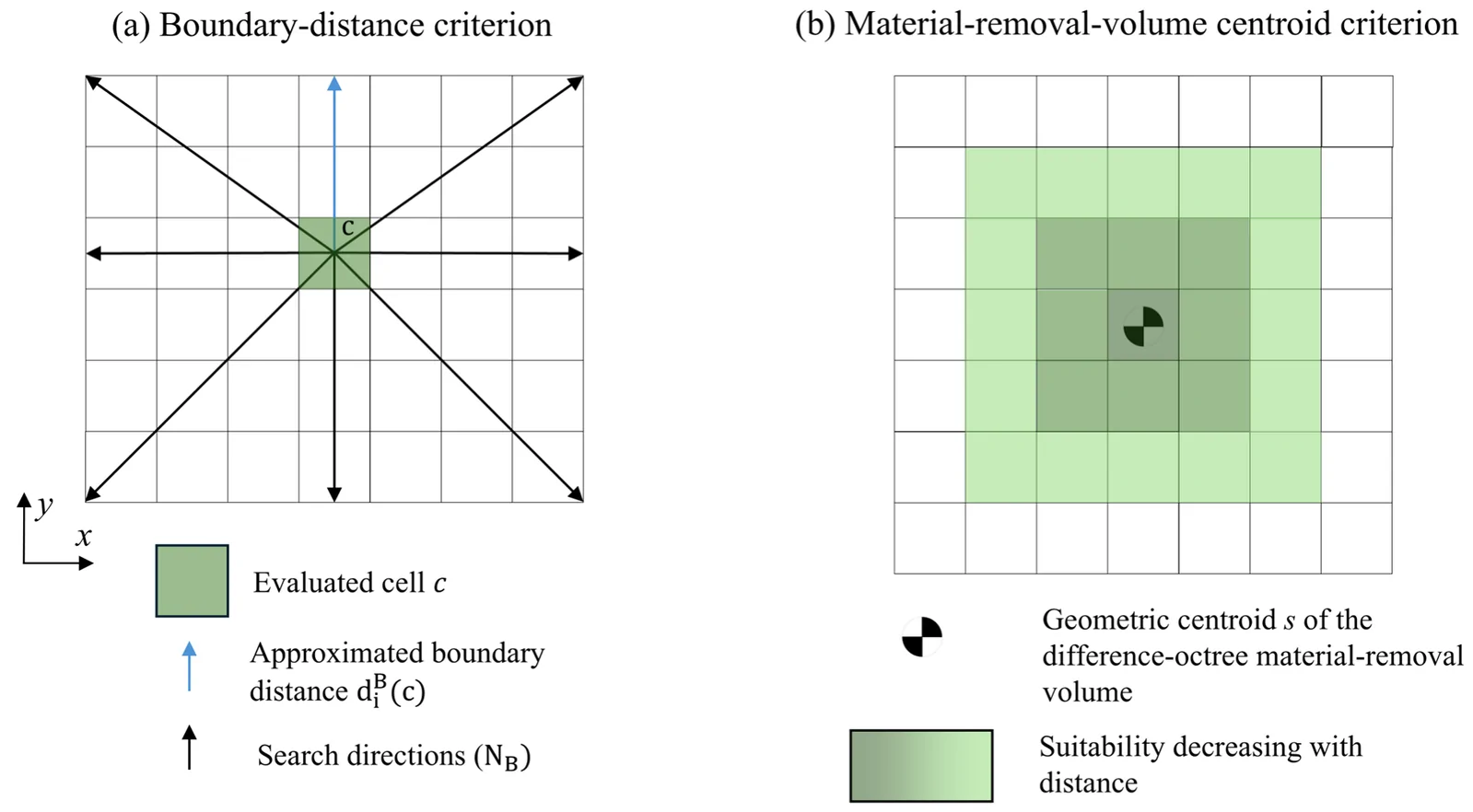
Geometric criteria for the fixturing suitability map.

**Figure 7:**
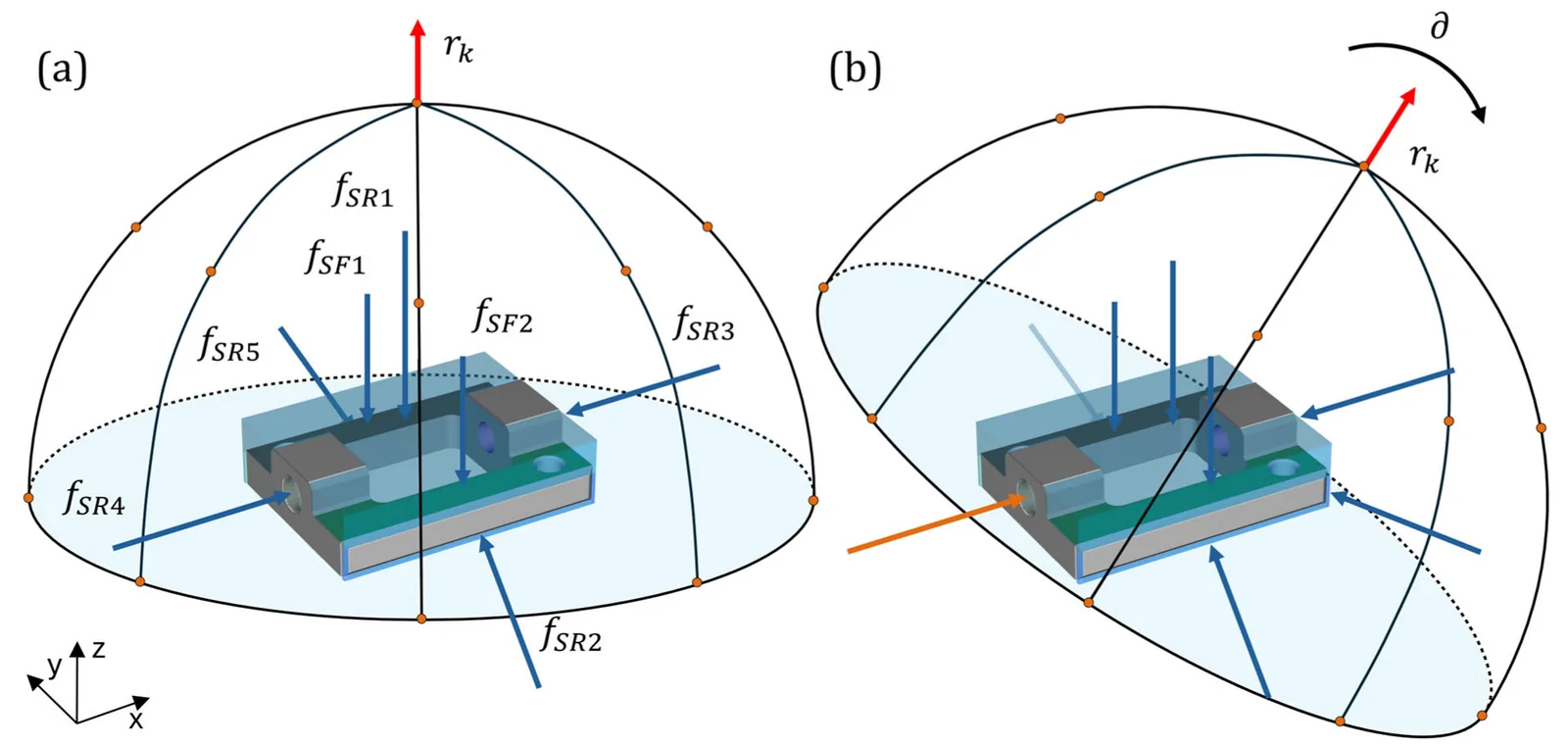
Table-orientation sampling and tool-access-direction compatibility: (**a**) initial orientation and (**b**) rotation by angle *∂*. Based on [11].

**Figure 8:**
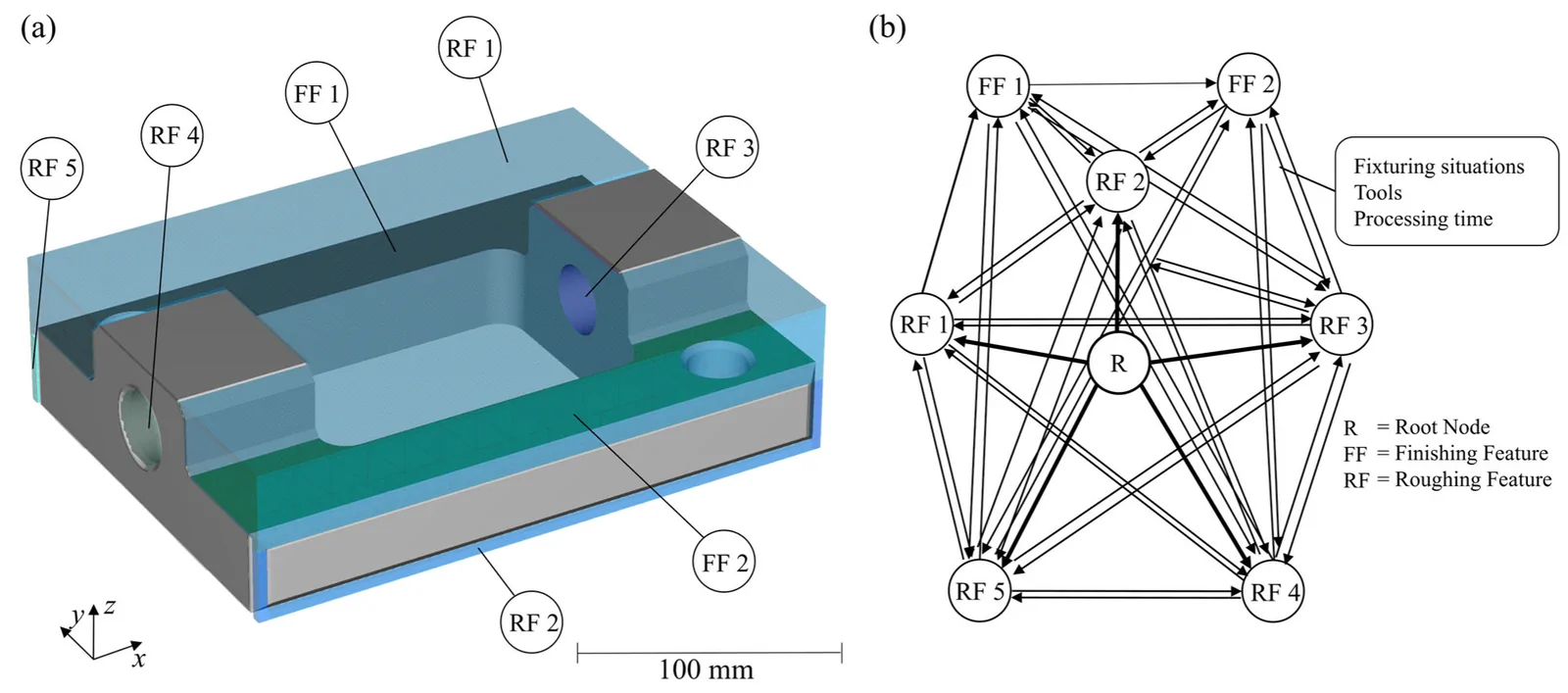
Example machining features (**a**) and corresponding operation-sequence graph with a highlighted admissible process-plan path (**b**), based on [11].

**Figure 9:**
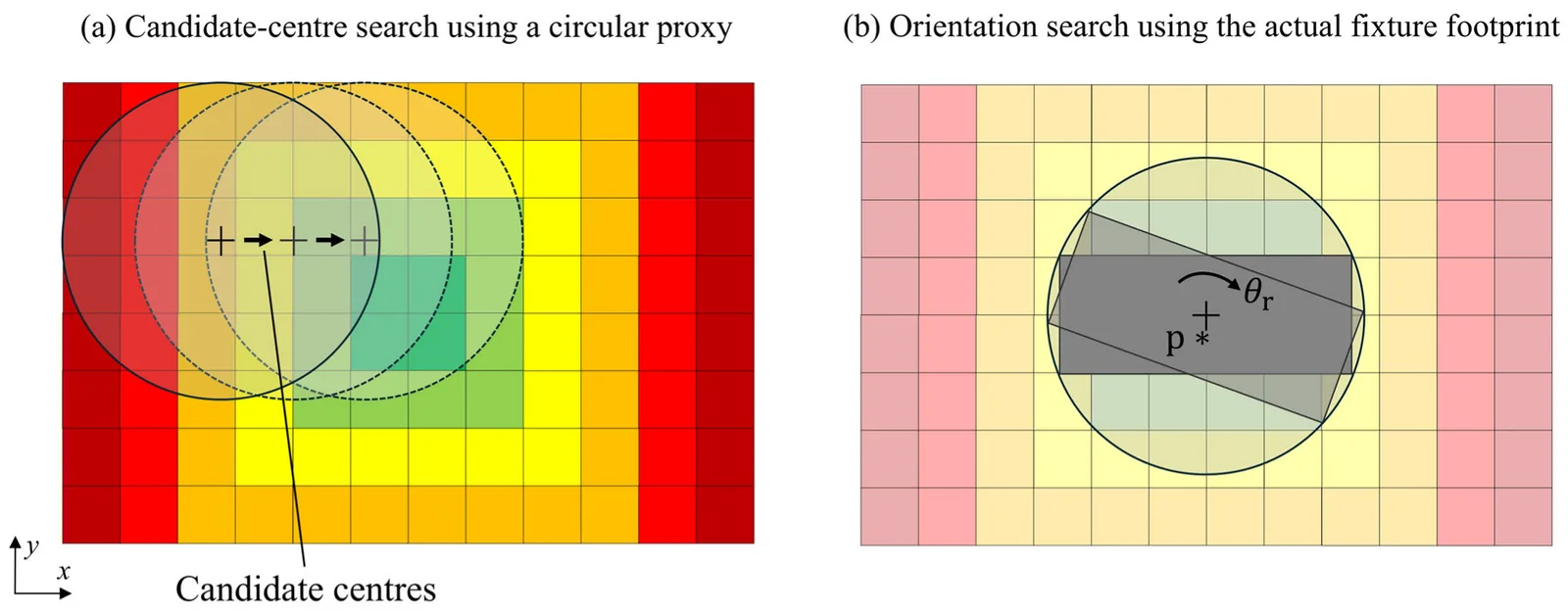
Two-stage geometric fixture-pose optimisation Colours indicate geometric suitability (red: low, green: high), circles represent candidate positions, and the selected centre (*p*∗) is used for orientation optimisation.

**Figure 10:**
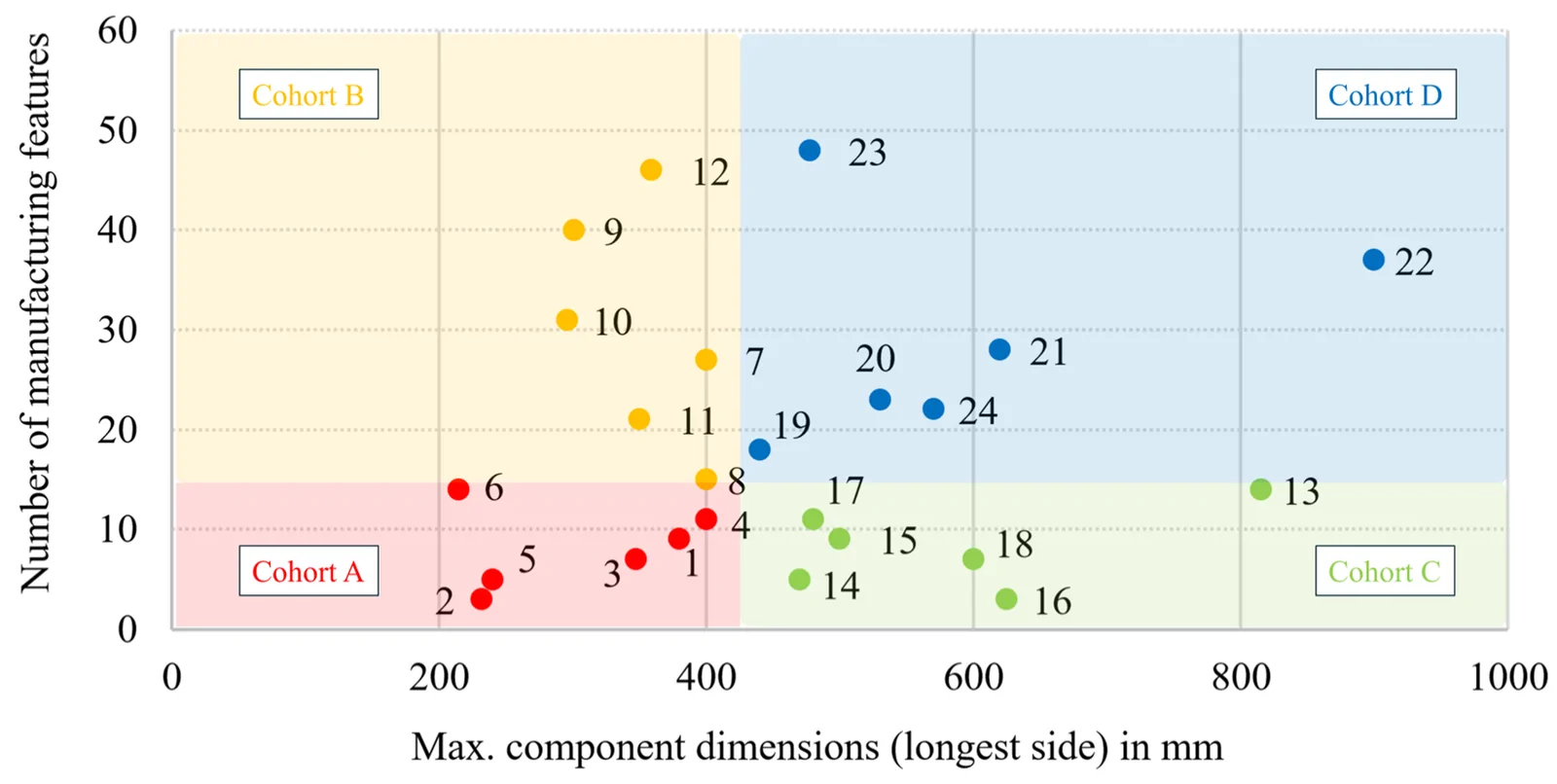
Distribution of the 24 benchmark parts according to maximum workpiece dimension and number of machining features. The four parts used for detailed analysis (2, 10, 13, and 23) belong to Cohorts A–D, respectively.

**Figure 11:**
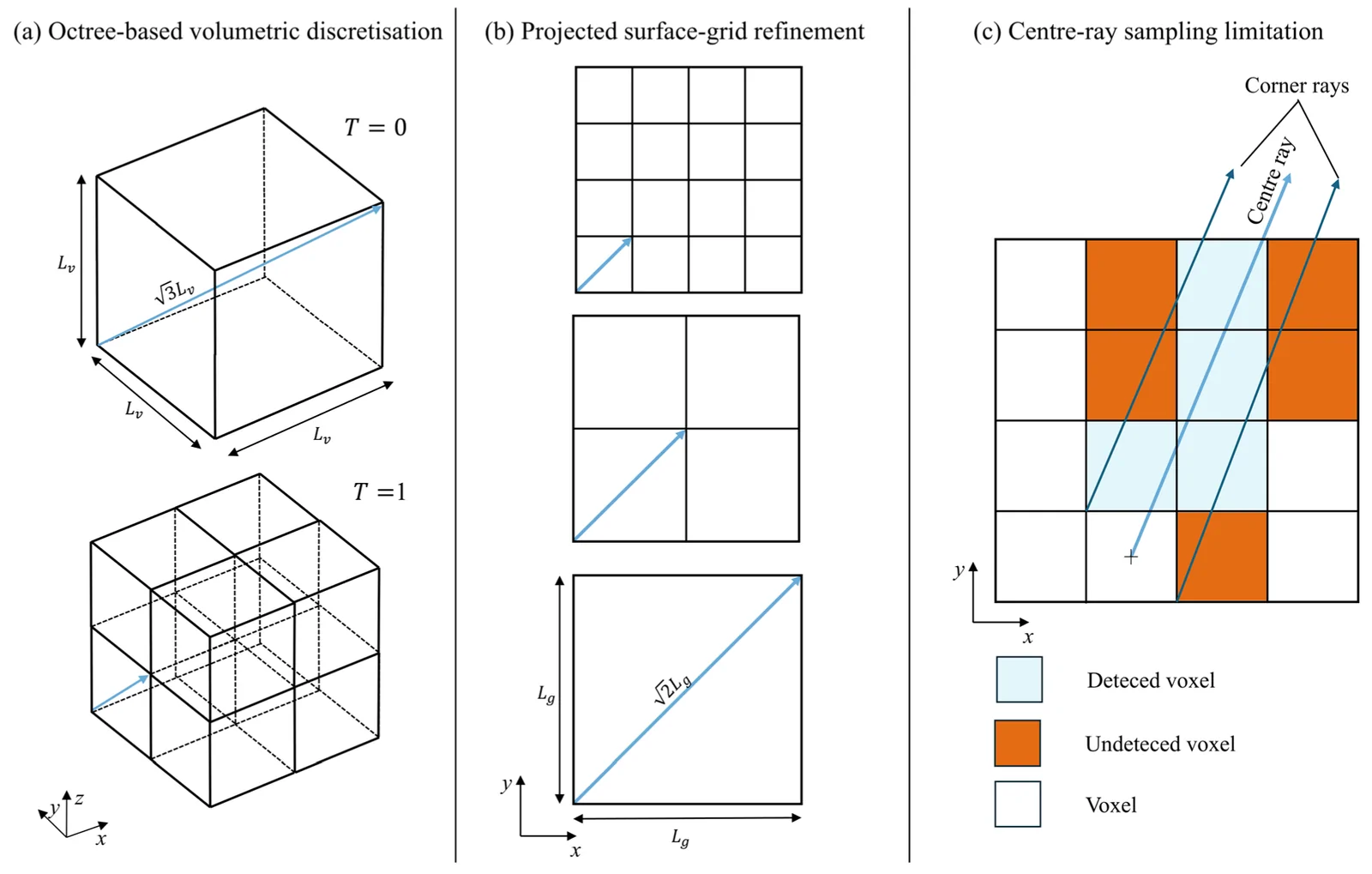
Geometric accuracy considerations for the volumetric representation, projected surface grid, and centre-ray accessibility analysis.

**Figure 12:**
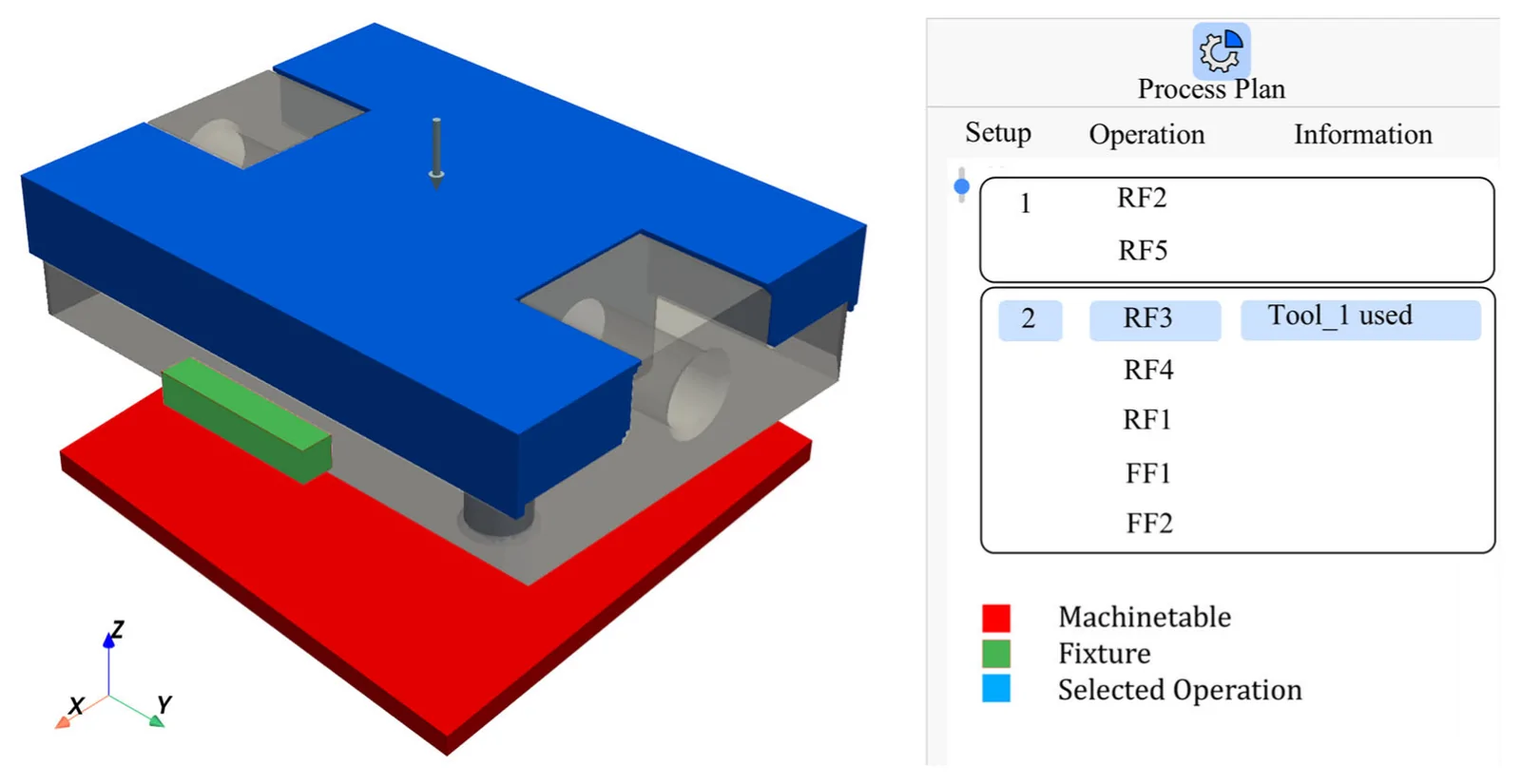
Demonstrator used for expert assessment, showing the selected operation with machining state, fixturing configuration, process plan, and tool information. Based on [11].

**Figure 13:**
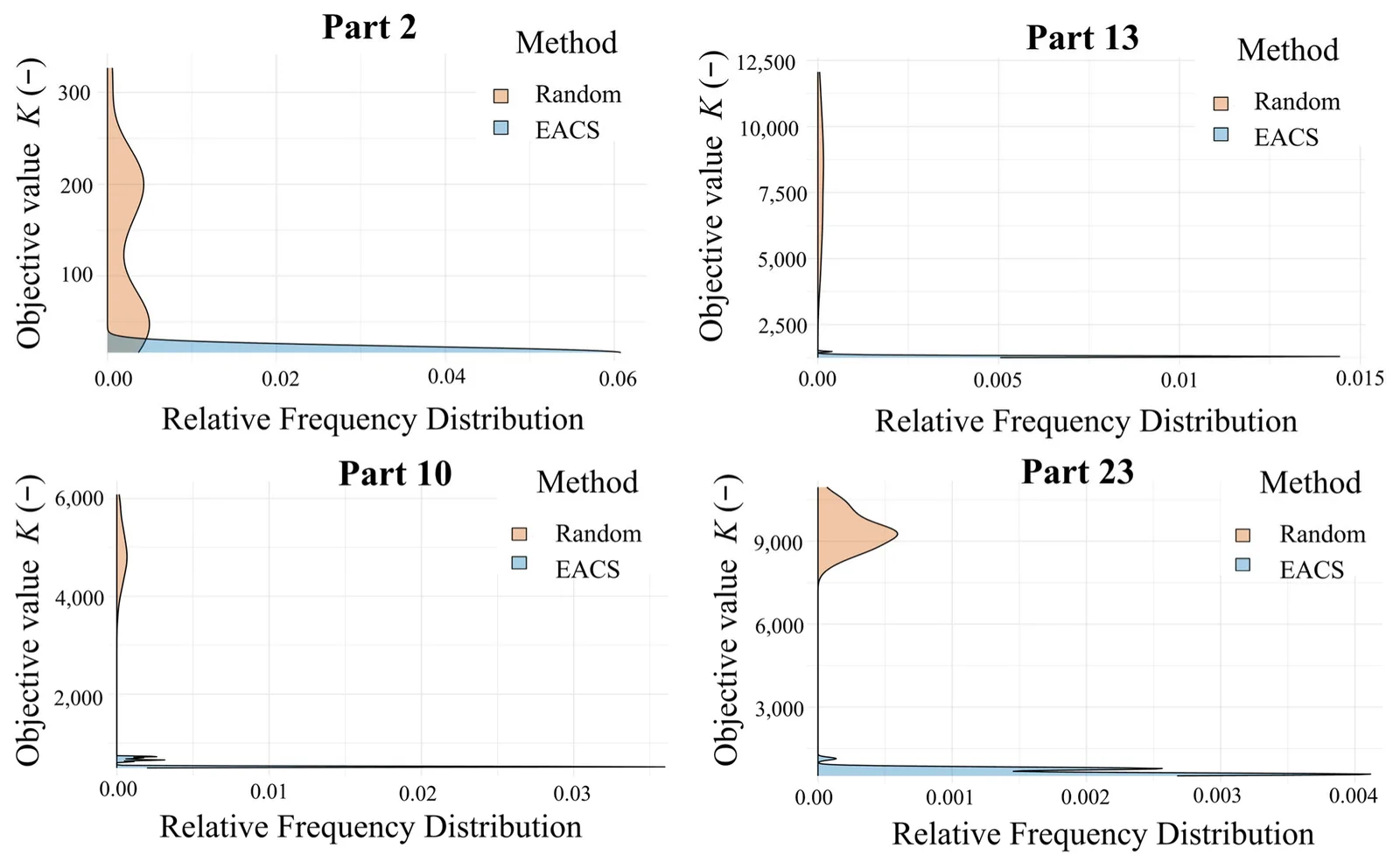
Empirical objective-value distributions for EACS and random admissible sampling of Parts 2, 10, 13, and 23.

**Figure 14:**
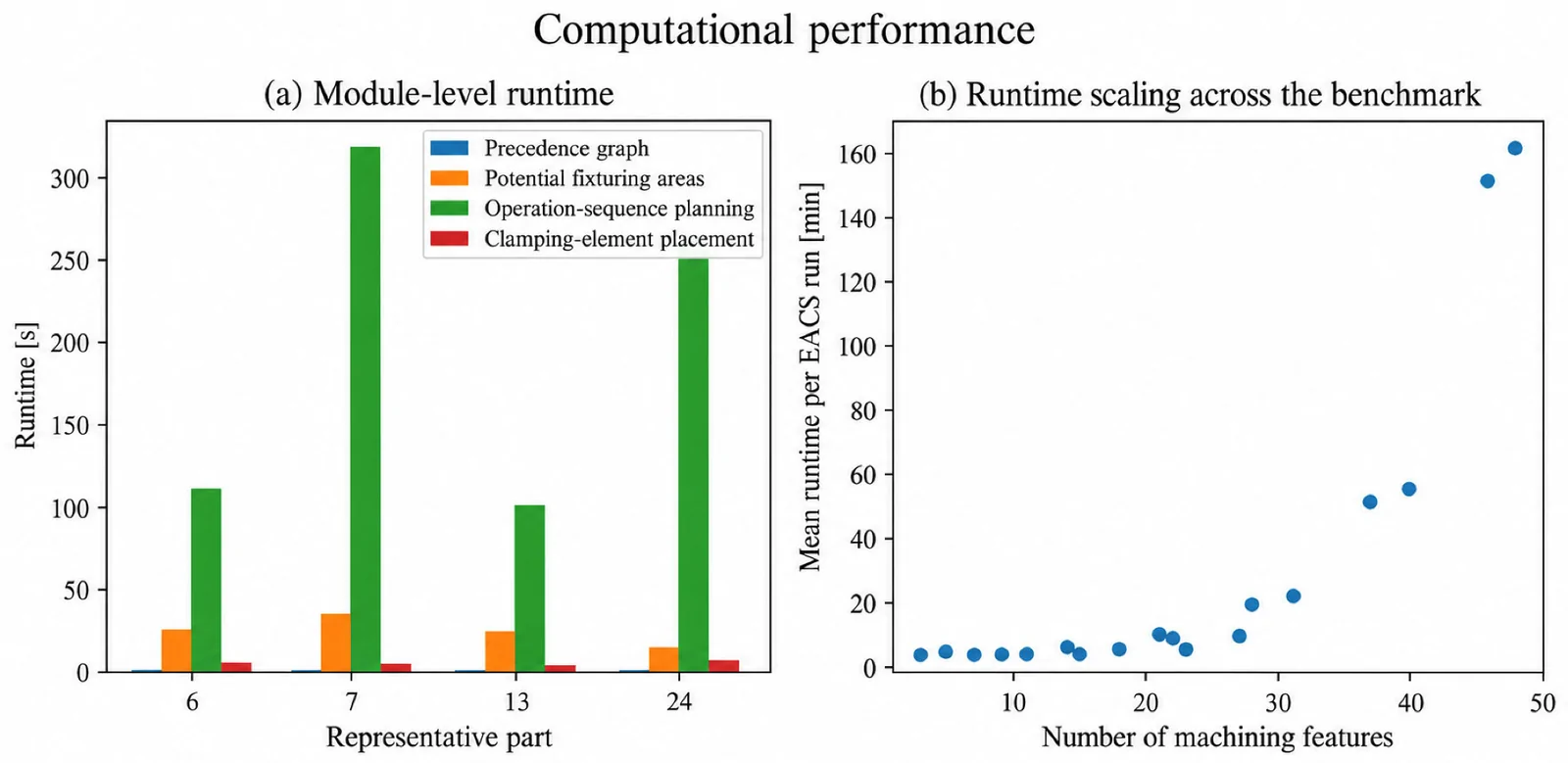
Computational performance of the planning framework: (**a**) module-level runtimes for the four representative benchmark parts and (**b**) mean EACS operation-sequence-planning runtime as a function of machining-feature count across all 24 parts. The corrected 27-feature count is used for Part 7.

**Table 1:** Positioning of the proposed method relative to selected process-planning and fixturing approaches.

Approach	Geometry-Derived Precedence	Intermediate States	Geometry-Derived Fixturing	State-Dependent Fixturing	Sequence Representation
Šormaz and Khoshnevis [6]	Yes, feature interactions	Geometry updated during sequencing	Not addressed	No	Feature-precedence network and AND–OR graph
Wang et al. [7]	Predefined constraints	No	No; setup alternatives represented	No	Directed process-planning graph
Li et al. [30]	Removal order geometrically represented	Yes	Not addressed	No	Subtraction field
Milosevic et al. [19]	Predefined in operation-precedence graph	No	No; setup changes considered in objective	No	Operation-precedence graph
Manafi and Nategh [34]	Geometric and technological rules	No	Concurrent setup and fixture planning	Not from evolving geometry	Integrated rule-based model
Šormaz et al. [31]	Feature interactions via feature-precedence network	Yes, for selected sequence	Used for downstream validation	Not part of sequence generation	Feature-based process planning
Zhang et al. [8]	Relations encoded in graph	Processing state represented	Not addressed	No	Attributed graph and Markov decision process
Dannen et al. [32]	Feature-independent accessibility	Yes	Clamping identified for voxel state	Yes	Two learned decision models
Wakhare et al. [42]	Feature-precedence network and tolerance precedence	No explicit material state	Setup formation from tool-access directions and geometric/dimensional tolerances	No	Rule-based setup model
Liu and Wang [24]	Yes, interacting-feature relations	No explicit state model	Not addressed	No	Machining-precedence graph
Mokhtar and Xu [25]	Yes, local and global precedence	No explicit state model	Not addressed	No	Feature-precedence graph
Tseng and Liu [20]	Predefined/feature relations	No	Fixturing setups considered concurrently	No	Sequence and setup analysis
Proposed	Derived from geometric accessibility	Yes	Derived from state-resolved geometry	Yes	State-dependent feasible planning space with dynamic operation release

**Table 2:** Comparison of the precursor study and the present work.

Aspect	Precursor Study [11]	Present Study
Geometry-derived precedence	Accessibility analysis and formal precedence graph	Integration of precedence constraints into explicit planning admissibility conditions
Evolving workpiece states	Intermediate material and surface states considered during planning	Unified state representation and explicit state-transition formulation
Fixturing alternatives	Geometry-based identification and evaluation of potential fixturing situations	Formal representation of state-dependent fixturing availability and admissibility
Process-plan representation	Operation graph integrating precedence and manufacturing resources	Explicit, solver-independent definition of the feasible planning space
Optimisation	EACS-based search with constraint-aware initialisation	Application of the established EACS approach to the formally defined planning space
Evaluation	Original 24-part benchmark and computational experiments	Additional consistency analyses, statistical reanalysis of archived benchmark results, and exploratory parameter-sensitivity experiments.

**Table 3:** System boundary of the proposed methodology.

Aspect	Included in the Present Method	Outside the Current System Boundary
Machining domain	Indexed 3 + 2-axis milling	Simultaneous five-axis path optimisation
Feature model	Predefined roughing (RFs) and finishing features (FFs)	Automatic feature recognition and alternative decomposition
Tool access	One predefined tool-access direction (TAD) per feature	Selection among alternative TADs; continuous tool-axis optimisation
Fixturing	Geometry-based candidate situations and geometric placement	Cutting-force, stiffness and deformation analysis
Machine representation	Abstract table orientation and geometric accessibility	Complete machine-tool kinematics and tool-holder motion
Output	Process plan and resource assignments	Final collision-free NC code

**Table 4:** Experimental parameterisation used for the computational evaluation.

Parameter Group	Parameter	Value Used in Evaluation
Geometric discretisation	Maximum octree depth *T*	7
Geometric discretisation	Surface-grid edge length *L_g_*	*L*_max_/100
Fixturing suitability	Boundary-distance search directions *N_B_*	16
Fixturing suitability	Suitability weights *w*_1_,*w*_2_	0.5, 0.5
Surface-pair generation	Normal-opposition tolerance *ε_n_*	1∙10^−5^
Surface-pair generation	Minimum projected overlap *A*_min_	756 mm^2^
Surface-pair generation	Relative separation parameter *λ_d_*	0.5
Table orientation	Angular sampling *N*_tab,1_ × *N*_tab,2_	16 × 16 index grid
Resource-space reduction	Retained fixturing situations *N_S_*_,max_	20 per feature
Clamping-element placement	In-plane orientations *N_R_*	16 unique orientations
Objective function	Setup-change weight *w_S_*	200
Objective function	Tool-change weight *w_T_*	10
EACS search	Pheromone exponent *α*	5
EACS search	Heuristic exponent *β*	0.4
EACS search	Ants per generation *N_A_*	100
EACS search	Maximum generations *N_G_*	100
EACS search	Pheromone-update parameter *ρ*	0.6
EACS initialisation	Proto-Ant initial solutions *N_P_*	10
EACS evolution	Selected-parent share *p*_sel_	0.2
EACS evolution	Tool-assignment mutation probability *p_T_*	0.9
EACS evolution	Fixturing-assignment mutation probability *p_S_*	0.1
EACS evolution	Start-operation mutation probability *p*_0_	0.1
EACS initialisation	Initialisation strategy	Constraint-aware Proto-Ants

**Table 5:** Cohort structure of the test-part basis.

Cohort	Maximum Dimension	Machining Features	Part IDs
A	≤400 mm	3–14	1–6
B	≤400 mm	15–50	7–12
C	401–1000 mm	3–14	13–18
D	401–1000 mm	15–50	19–24

**Table 6:** Professional background of the experts participating in the qualitative validation.

Expert Role	CAM Experience	Machining Environment
CNC team leader	29 years	5-axis
Process preparation	12 years	5-axis
Workshop manager	30 years	3-axis and 5-axis
CAM planner and machinist	13 years	3-axis and 5-axis

**Table 7:** Clamping-element placement versus exhaustive admissible-pose enumeration. Data from [11].

Part	Fixturing Situations	Mean Rank	Mean Enumerated Poses	Mean Deviation from Reference Optimum
6	2	24.00	793.0	0.76%
7	3	39.67	1651.0	0.67%
13	4	130.00	2960.5	6.39%
24	4	6.75	455.0	0.77%

**Table 8:** Across-part comparison of EACS and random admissible planning.

Statistic	Result
Test parts	24
EACS optimisation runs per part	58
Random admissible samples per part	1000
Parts with lower mean EACS objective	24/24
Parts with complete observed separation	23/24
Directional success proportion	1.000
Exact 95% confidence interval	0.858–1.000
Exact two-sided sign-test p	1.19·10^−7^
Mean probability of superiority	0.99933
Minimum probability of superiority	0.9840

**Table 9:** Characteristics of the four benchmark parts used as illustrative cases for detailed analysis.

Part (Case)	Dimensions [mm]	RF/FF	n	TADs	Unconstrained Orders n!	*A_i_*
2 (A)	232 × 205 × 55	1/2	3	1	6	0.9840
10 (B)	295.8 × 119 × 119	13/18	31	3	8.22 × 10^33^	1.0000
13 (C)	437.5 × 440 × 815	4/10	14	5	8.72 × 10^10^	1.0000
23 (D)	477.5 × 292.5 × 145	16/32	48	3	1.24 × 10^61^	1.0000

**Table 10:** State-admissible fixturing candidates under different retention limits. Counts are aggregated across selected feature–state combinations, with candidate truncation preceding state-dependent filtering.

Part (Case)	Without Truncation	NS, max = 10	NS, max = 20	NS, max = 30
2 (A)	4	4	4	4
10 (B)	583	331	512	581
13 (C)	483	199	362	444
23 (D)	3515	1123	2591	2703

## Data Availability

The original contributions presented in this study are included in the article/Supplementary Materials. Further inquiries can be directed to the corresponding author.

## Supplementary Materials

The following supporting information can be downloaded at https://www.mdpi.com/article/10.3390/ma19214444/s1. Table S1: Complete benchmark definitions and part-level evaluation characteristics; Data S1: Run-level results of the computational benchmark, including 58 EACS optimisation runs and 1000 random admissible samples for each of the 24 benchmark parts (Microsoft Excel file).
