# Supplementary material for: Geometry-Based Design of a State-Dependent Process-Planning Space with Integrated Fixturing for 3 + 2-Axis Milling
Source: Materials (Basel). 2026 Oct 22;19(21):4444. doi: 10.3390/ma19214444 (PMC13764177; doi:10.3390/ma19214444)
Supplement: Supplementary file 1 [file materials-19-04444-s001.zip › materials-4612802-Supplementary Table S1.pdf]

<u>Supplementary Table S1. Complete 24-Part Test Basis.</u>

Part	Dimensions [mm]	Features	RF	FF	TADs	Fixturing situations	A_i
1	380 × 125 × 120	9	4	5	3	11	1.0000
2	232 × 205 × 55	3	1	2	1	10	0.9840
3	347.5 × 347.5 × 119	7	4	3	2	17	1.0000
4	400 × 235 × 95	11	1	10	1	15	1.0000
5	93 × 240 × 150	5	3	2	3	20	1.0000
6	160 × 215 × 55	14	9	5	9	22	1.0000
7	360 × 150 × 400	27	9	18	6	24	1.0000
8	400 × 45 × 400	15	7	8	6	4	1.0000
9	301 × 203 × 147	40	10	30	4	23	1.0000
10	295.8 × 119 × 119	31	13	18	3	37	1.0000
11	35 × 350 × 350	21	21	0	2	12	1.0000
12	359.125 × 178.5 × 144.5	46	11	35	4	58	1.0000
13	437.5 × 440 × 815	14	4	10	5	28	1.0000
14	470 × 225 × 125	5	3	2	3	13	1.0000
15	500 × 100 × 350	9	5	4	4	15	1.0000
16	625 × 75 × 340	3	1	2	1	15	1.0000
17	480 × 290 × 60	11	7	4	4	20	1.0000
18	600 × 70 × 600	7	4	3	2	18	1.0000
19	240 × 439.9 × 360	18	9	9	6	32	1.0000
20	294.7 × 250.5 × 530	23	13	10	7	18	1.0000
21	620 × 200 × 280	28	21	7	6	28	1.0000
22	87.5 × 225 × 900	37	8	29	4	23	1.0000
23	477.5 × 292.5 × 145	48	16	32	3	93	1.0000
24	570 × 101.25 × 250	22	7	15	6	17	1.0000
